# An injectable bioceramics-containing composite hydrogel promoting innervation for pulp-dentin complex repair

**DOI:** 10.1038/s41368-025-00398-0

**Published:** 2025-10-01

**Authors:** Xingyu Tao, Hongjian Zhang, Peng Mei, Jinzhou Huang, Bing Fang, Zhiguang Huan, Chengtie Wu

**Affiliations:** 1 State Key Laboratory of High Performance Ceramics, Shanghai Institute of Ceramics, Chinese Academy of Sciences, Shanghai, China; 2 Center of Materials Science and Optoelectronics Engineering, University of Chinese Academy of Sciences, Beijing, China; 3 Department of Orthodontics, Shanghai Ninth People’s Hospital, Shanghai Jiao Tong University School of Medicine, College of Stomatology, Shanghai Jiao Tong University, National Center for Stomatology, National Clinical Research Center for Oral Diseases, Shanghai Key Laboratory of Stomatology, Shanghai Research Institute of Stomatology, Shanghai, China

**Keywords:** Biomedical materials, Biomineralization

## Abstract

Dental pulp-dentin complex defects remain a major unresolved problem in oral medicines. Clinical therapeutic methods including root canal therapy and vital pulp therapy are both considered as conservative strategies, which are incapable of repairing the pulp-dentin complex defects. Although biomaterial-based strategies show remarkable progress in antibacterial, anti-inflammatory, and pulp regeneration, the important modulatory effects of nerves within pulp cavity have been greatly overlooked, making it challenging to achieve functional pulp-dentin complex regeneration. In this study, we propose an injectable bioceramics-containing composite hydrogel in combination of Li-Ca-Si (LCS) bioceramics and gelatin methacrylate matrix with photo-crosslinking properties. Due to the sustained release of bioactive Li, Ca and Si ions from LCS, the composite hydrogels possess multiple functions of promoting the neurogenic differentiation of Schwann cells, odontogenic differentiation of dental pulp stem cells, and neurogenesis-odontogenesis couples in vitro. In addition, the in vivo results showed that LCS-containing composite hydrogel can significantly promote the pulp-dentin complex repair. More importantly, LCS bioceramics-containing composite hydrogel can induce the growth of nerve fibers, leading to the re-innervation of pulp tissues. Taken together, the study suggests that LCS bioceramics can induce the innervation of pulp-dentin complex repair, offering a referable strategy of designing multifunctional filling materials for functional periodontal tissue regeneration.

## Introduction

Teeth are one of the most important tissues of the human body. However, due to bacterial invasion and trauma, dental pulp necrosis and dentin defects can occur, resulting in the deterioration of tooth structures and the compromise of physiological functions.^1^ Dental caries is a pertinent example. Severe forms of this condition can lead to damage to the dentin matrix, thus exposing the dental pulp to the oral microenvironment. The dental pulp is susceptible to infection by microorganisms, which can result in inflammation and necrosis.^2–4^ In clinical, the treatment of pulp-related caries commonly involves the utilization of medical pastes or pulp capping, avoiding further inflammation and bacterial invasion.^5^ However, when the dental pulp was seriously infected or necrotic, the therapeutic approach entails root canal therapy (RCT), which uses inert materials to replace the necrotic tissue.^6,7^ It should be noted that the currently applicable filling materials such as calcium hydroxide, exhibit a limited ability to promote odontogenic differentiation of dental pulp cells and dentin formation.^6,8^ The long-term delayed regeneration and structure integration of pulp-dentin complex can increase the brittleness of teeth, loss of defense ability and teeth vitality.^7^ Generally, dentin caries is associated with simultaneous damage to the dentin and pulp. Moreover, due to the close structural connection and interdependent functions between dental pulp and dentin,^9,10^ the concept of integration regeneration for pulp-dentin complex has attracted increasing attention.^2,11^

Dental pulp-dentin complex is densely innervated with multiple nerve fibers, that discriminate the functions of sense external stimuli such as pain and temperature.^7,12^ Besides, sensory nerves could secret numerous neurotransmitters and neurotrophic factors to regulate inflammation, angiogenesis and the odontogenic differentiation of dental pulp cells, thus actively participating in pulp-dentin complex repair.^7,13–15^ In the sensory nerve-deficient microenvironment, the supplementation of activin B has been confirmed to promote the proliferation and reduced the apoptosis of dental pulp stem cells (DPSCs).^15^ It is reported that Schwann cells (SCs) possess the capacity of secreting extracellular vesicles to induce angiogenesis and neurite outgrowth.^6,16,17^ Moreover, SCs-derived extracellular vesicles can also activate SDF-1/CXCR4 axis to recruit endogenous stem cells, and thus contribute to the formation of structures similar to the pulp-dentin complex.^6,16^ Hence, innervation should be fully considered when designing biomaterials for dental pulp-dentin complex regeneration. Unfortunately, to our knowledge, current strategies are mainly focused on antibacterial,^18,19^ immune regulation,^20,21^ and mineral deposition,^22–24^ which largely ignored the great significance of innervation, leading to unsatisfactory therapeutic outcomes. Therefore, it remains challenging to achieve innervated pulp-dentin complex regeneration.^7,25^

Silicate bioceramics have gained increasing attention in regenerative medicine owing to their excellent biocompatibility and biodegradability,^26,27^ and have been successfully employed for tissue regeneration, including bone, cartilage, skin, muscles, and nerve tissues.^28–32^ The bioactive ions released from silicate bioceramics could create beneficial ionic microenvironments for regulating multiple cell behaviors, including proliferation, migration and differentiation.^32–35^ Lithium (Li), one of the essential trace elements, possesses notable neuroactive and neuroprotective effects.^36–38^ Studies have shown that Li ions enhance the migration, differentiation and myelination of SCs via β-catenin signals.^39,40^ Moreover, Li ions could promote the neuronal differentiation of neural stem cells by activating the PI3K-AKT signaling pathway.^32^ It is reported that Li-containing fillers could stimulate DPSCs migration and odontogenic differentiation through activating Wnt/β-catenin pathway, inducing dentin formation.^41^ Besides, as the main component of tooth, calcium (Ca) actively participates in mineral deposition and dentin formation.^42^ Ca ions also play crucial roles in neurogenesis.^43,44^ Moreover, silicon (Si) ions have been demonstrated to induce the osteogenic differentiation of bone mesenchymal stem cells, and promote the neurite outgrowth of dorsal root ganglion neurons as well as secreting neuropeptides.^45,46^ Hence, it is reasonable to speculate that Li-Ca-Si (LCS) bioceramics could act as bioactive factors to induce neurogenesis odontogenesis, which further promote innervated pulp-dentin complex regeneration.

**Fig. 1:**
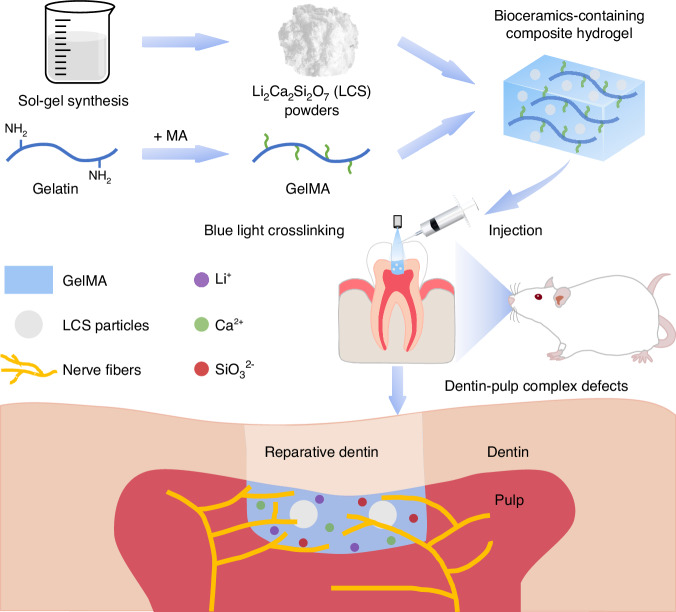
Schematic diagram of the preparation of an injectable bioceramics-containing composite hydrogel and its application in the regeneration of innervated dentin-pulp complex. The injectable composite hydrogel was prepared by incorporating lithium calcium silicate (LCS) bioceramics into GelMA hydrogel. The bioceramics-containing composite hydrogel was in situ injected and then crosslinked by blue light to fill the defects. Multiple bioactive Li, Ca and Si ions released by the composite hydrogel could promote odontogenesis and neurogenesis, thus contributing to the regeneration of dentin-pulp complex with innervation

Herein, we successfully prepared an injectable bioceramics-containing composite hydrogel composed of LCS bioceramics particles and gelatin methacryloyl (GelMA) matrix for the treatment of pulp-dentin complex defects (Fig. 1). Firstly, GelMA was chosen as a filler for in situ injected into the defects in a minimally invasive manner, serving as a barrier to protect pulp tissues. Secondly, LCS bioceramic particles were incorporated into the hydrogel, functioning as bioactive factors for regulating cell behavior. Specifically, the released bioactive Li, Ca, Si ions have the capacity to enhance the migration, and neurogenic differentiation of SCs, as well as the odontogenic differentiation of DPSCs. Besides, the function of composite hydrogel in mediating neurogenesis-odontogenesis couples was investigated. Finally, the effectiveness of bioceramics-containing composite hydrogel on innervated pulp-dentin complex regeneration was explored in vivo. Overall, we expect that this study could offer a new concept for achieving functional pulp-dentin regeneration via inducing innervation by biomaterials.

## Results

### Preparation and characterization of LCS bioceramics and injectable composite hydrogel

**Fig. 2:**
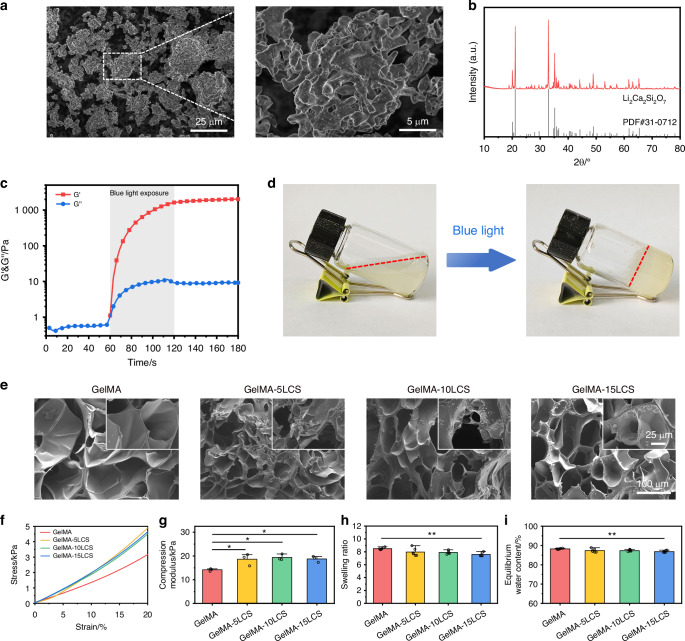
Characterization of Li_2_Ca_2_Si_2_O_7_ (LCS) bioceramics particles and bioceramics-containing composite hydrogel. **a** SEM image of LCS particles. **b** XRD pattern of LCS particles. **c** Modulus-time curves of composite hydrogel with blue light crosslinking. **d** Digital photographs of hydrogel before and after crosslinked by blue light. **e** SEM images of these composite hydrogels. **f** The stress-strain curve of composite hydrogels. **g** The compression modulus analysis of composite hydrogels (*n* = 3). **h** Swelling ratio of the hydrogels (*n* = 6). **i** Equilibrium water content of the hydrogels (*n* = 6). *P < 0.05, **P < 0.01, ***P< 0.001. High-purity LCS bioceramics particles and bioceramics-containing composite hydrogels were successfully prepared, and the incorporation of LCS particles could enhance the mechanical characteristics of hydrogels

LCS bioceramics particles were synthesized by sol-gel method as our previous report.^33,47^ As shown in Fig. 2a, LCS particles exhibited an irregular morphology with micron-scale size. The XRD pattern showed that all the diffraction peaks of particles could be well indexed into Li_2_Ca_2_Si_2_O_7_ phase (Fig. 2b). Fig. S1 showed that the particle size of LCS bioceramics particles is below 50 μm. GelMA hydrogel was synthesized according to previous studies.^45,48^ As shown in Fig. 2c, the storage modulus (G’) of the hydrogel was immediately increased and significantly higher than loss modulus (G”) after exposed to blue, indicating its light-triggered gelation properties. Figure 2d showed the representative images of sol-gel transition process of composite hydrogel.

To form the bioceramics-containing composite hydrogel, different amounts of LCS particles were incorporated into the GelMA solution. Macroscopic photographs showed that the transparency of hydrogel decreased, turning from translucent to white with an increase in LCS particles concentrations (Fig. S2). As demonstrated in Fig. 2e, all hydrogels exhibited an interconnected macro-porous network structure. LCS particles were uniformly distributed in the wall of hydrogels without obvious agglomerations (Fig. S3). The XRD pattern of Fig. S4 showed that LCS bioceramic particles are loaded into the GelMA hydrogel matrix. Furthermore, the rheology and mechanical properties of composite hydrogels were tested. The results of the strain sweep experiment indicated that the G” values were obviously lower than the G’ values at lower strain levels, suggesting the stable solid state of these hydrogels (Fig. S5). The critical strain (corresponding to the intersection of G’ and G”) was approximately 290%, 290%, 250%, and 180% in the GelMA, GelMA-5LCS, GelMA-10LCS and GelMA-15LCS, respectively. Figure 2f, g showed the stress-strain curves and the corresponding compression modulus calculated from the slope of 0–10% strains of the curves of these hydrogels. The compression modulus of GelMA, GelMA-5LCS, GelMA-10LCS and GelMA-15LCS were (14.16 ± 0.54) kPa, (18.63 ± 2.49) kPa, (19.36 ± 1.30) kPa, and (18.73 ± 1.28) kPa, respectively. It could be found that the incorporation of LCS bioceramics slightly enhanced the compression modulus of the composite hydrogel as compared with pure GelMA hydrogel, while these three LCS-containing composite hydrogels exhibited non-significant differences in statistics. Owing to its interconnected networks structure, hydrogel possessed suitable swelling properties. As shown in Fig. 2h, i, all groups exhibited well-swelling and equilibrium water content (EWC) behaviors, while the values were slightly decreased with the increasing of LCS particles concentrations. Since physiological activities often need to be within a certain pH range to meet the requirement of biofunction, the effect of composite hydrogels on solution pH also needs to be paid attention. As shown in Fig. S6, the incorporation of LCS bioceramic could increase the pH value. After 21-day soaking periods, the pH of GelMA, GelMA-5LCS, GelMA-10LCS, and GelMA-15LCS was 7.40 ± 0.02, 7.92 ± 0.06, 9.20 ± 0.07, and 9.87 ± 0.14, respectively. Moreover, the degradation rates of GelMA, GelMA-5LCS, GelMA-10LCS, and GelMA-15LCS at 21 days were found to be 44.82% ± 3.70%, 30.40% ± 5.90%, 24.78% ± 5.36%, and 22.10% ± 2.90%, respectively (Fig. S7).

**Fig. 3:**
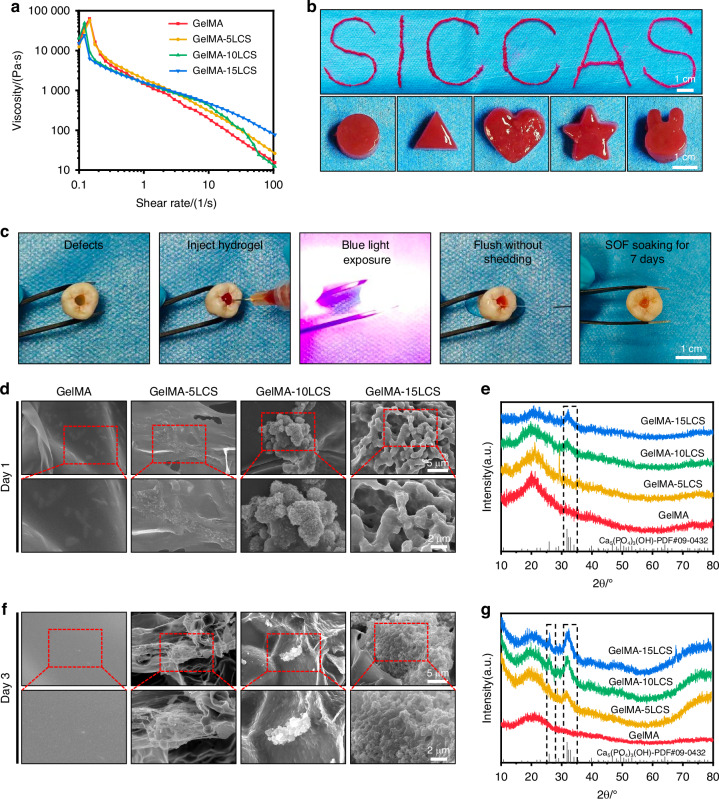
Injectability and in vitro mineralization properties of bioceramics-containing composite hydrogel. **a** The viscosity-shear rate curves of composite hydrogels with different concentrations of LCS particles. **b** Representative photographs of injectability characterizations. **c** Digital images showing the ability of composite hydrogels to fill pulp-dentin defects. **d** SEM images and **e** corresponding XRD pattern of composite hydrogels after immersed in SOF for 1 days. **f** SEM images and **g** corresponding XRD pattern of the composite hydrogels after immersed in SOF for 3 days. Bioceramics-containing composite hydrogels possessed satisfactory injectability and good mineralization properties

As shown in Fig. 3a, an inverse correlation between shear rate and viscosity was evident. The viscosity of the hydrogel exhibited a substantial decrease with the increase of shear rate. Besides, the oscillation frequency tests were performed under the range of 0.1-10 Hz. In this range, the G’ values consistently exceeded the G” values, showing its stable gel states (Fig. S8). Moreover, as demonstrated in Figs. 3a, S8, and S9, the incorporation of LCS bioceramic particles does not significantly affect the shear-thinning properties, frequency stability, and temperature sensitivity of GelMA hydrogels. Subsequently, the injectability behaviors of composite hydrogels were also verified by writing specified word ‘SICCAS’ from a syringe with 25G needle, and injected into molds with various shapes, such as circle, triangle, heart, pentagram and rabbit head (Fig. 3b). The composite hydrogel possessed suitable injectability and shape fidelity. Moreover, the simulation experiments were also performed to demonstrate the convenience of injectability in clinical. The composite hydrogel (stained with red dyes) was first filled into the dentin-pulp defects, and then crosslinked with blue light for 5 seconds. The composite hydrogel could be stably filled in the defect area even under the erosion of flush water, and maintained its stability after immersion in simulated oral fluid (SOF) (Fig. 3c). The above results showed that the rheological properties of bioceramics-containing composite hydrogels are similar to those of pure GelMA hydrogels.

As shown in Fig. 3d–g, there was no mineralization product generated from the pure GelMA hydrogel, and the surface remained smooth throughout the immersion period. After soaked in SOF for 1 day, mineralization products can be observed in GelMA-10LCS and GelMA-15LCS, which is confirmed by the characteristic peaks of hydroxyapatite of the XRD pattern (Fig. 3d, e). Mineralization products were also found in GelMA-5LCS group after being soaked in SOF for 3 days. Furthermore, an increase in the LCS content resulted in an enhancement of the intensity of the mineralization peak. (Fig. 3f, g).

### The neurogenic activity of bioceramics-containing composite hydrogel

**Fig. 4:**
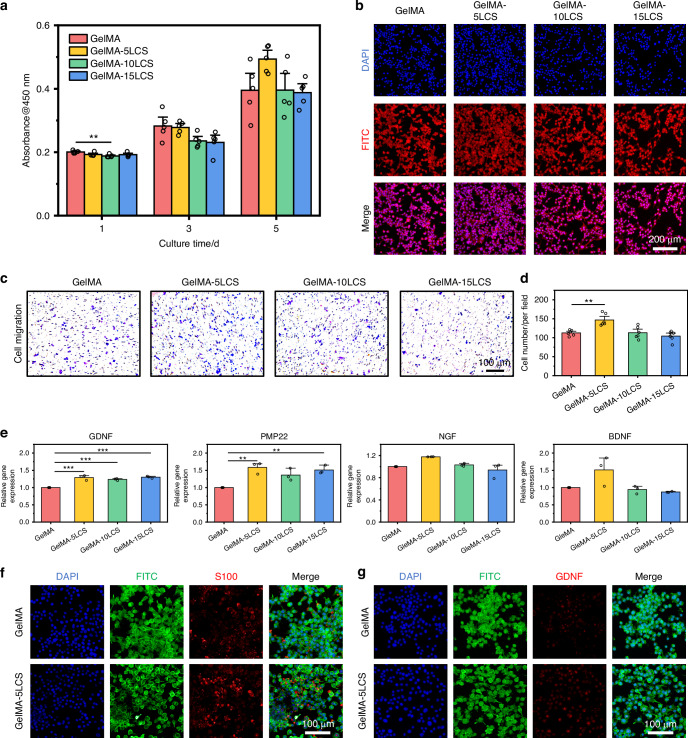
The neurogenic activity of bioceramics-containing composite hydrogels. **a** Proliferation of SCs cultured with composite hydrogels for 1, 3, and 5 days (*n* = 5). **b** Morphology of SCs on the surface of these hydrogels. **c** Crystalline violet staining images of the migrated SCs after cultured with composite hydrogels. **d** Quantitative statistics results of migrated SCs (*n* = 6). **e** Expression of neurotrophic factor and myelination-related genes in SCs cultured with composite hydrogels for 5 days (*n* = 3). Representative immunofluorescent images of **f** S100 and **g** GDNF proteins in SCs after cultured with GelMA and GelMA-5LCS hydrogel for 5 days. (Blue: cell nuclei; Green: cytoskeleton; Red: proteins). *P < 0.05, **P < 0.01, ***P < 0.001. GelMA-5LCS composite hydrogels could effectively enhance the proliferation, migration, and neurogenic differentiation activity of SCs

According to live/dead staining images and cell counting kit 8 (CCK-8) results shown in Fig. S10 and Fig. 4a, it could be observed that cells survived well in all the groups without significant differences between each other for 5 days of culture. Besides, the skeleton and nucleus of SCs were stained by FITC and DAPI, respectively, in order to observe cell adhesion. It can be observed that SCs spread well on the surface of composite hydrogels, confirming their favorable cytocompatibility (Fig. 4b). A Transwell migration assay was further conducted to evaluate the impact of the LCS-containing composite hydrogels on cell migration activities. As shown in Fig. 4c, a significantly higher number of migrated cells were observed in the GelMA-5LCS group in comparison to the other groups. The statistical analysis showed that the quantity of migrated cells in the GelMA-5LCS group was notably greater, suggesting that certain concentrations of LCS bioceramics could promote the migration activity of SCs (Fig. 4d). The expression of genes associated with neurogenesis in SCs was assessed using RT-qPCR experiments. After 5-days culture, the expression of GDNF, PMP22, NGF, and BDNF was significantly higher in the GelMA-5LCS group in comparison to those in GelMA group (Fig. 4e). Furthermore, immunofluorescence images (Fig. 4f, g) and semi-quantitative analysis (Fig. S11) indicated that the GelMA-5LCS composite hydrogel obviously promoted the expression of S100 and GDNF proteins in SCs as compared with GelMA hydrogels. Moreover, the concentrations of bioactive ions released from LCS bioceramics-containing composite hydrogel during cell culture are shown in Table S1. The composite hydrogel has been observed to facilitate the continuous release of bioactive ions (Fig. S12 and Table S1). Taken together, GelMA hydrogel containing 5% LCS bioceramics particles exhibited the best performance in the neurogenic differentiation of SCs.

### The odontogenic activity of bioceramics-containing composite hydrogel

**Fig. 5:**
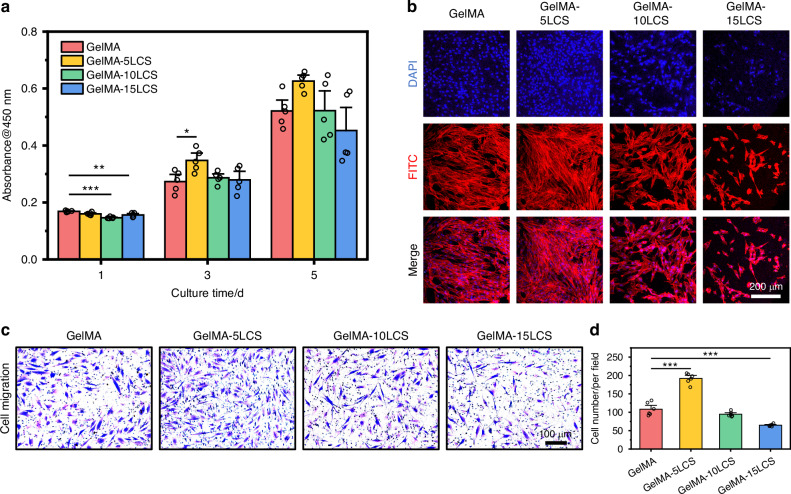
The proliferation and migration activities of DPSCs cultured with bioceramics-containing composite hydrogels. **a** Proliferation of DPSCs cultured with composite hydrogels for 1, 3 and 5 days (*n* = 5). **b** Morphology of DPSCs on the surface of these hydrogels. **c** Crystalline violet staining images of migrated DPSCs after cultured with these composite hydrogels. **d** Quantitative statistics of the migrated DPSCs (*n* = 6). *P < 0.05, **P < 0.01, ***P < 0.001. GelMA-5LCS composite hydrogels could obviously enhance the proliferation and migration of DPSCs

**Fig. 6:**
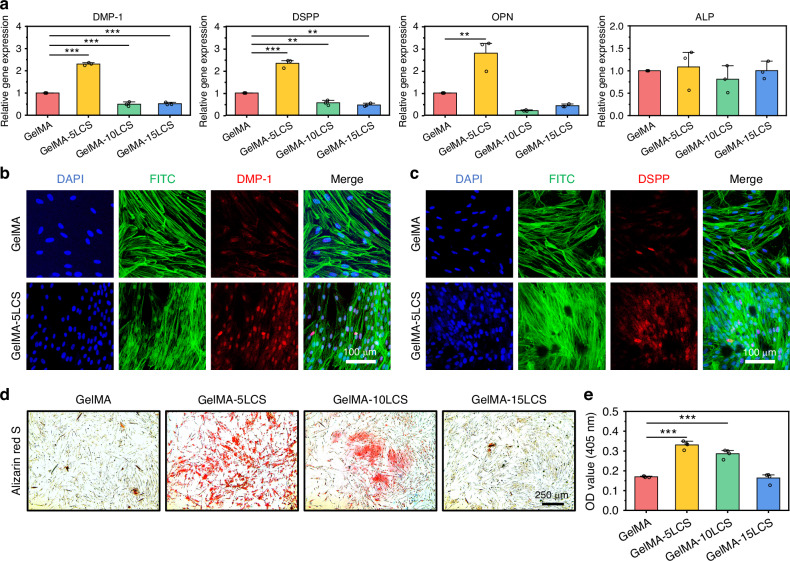
The odontogenic differentiation activity of DPSCs cultured with bioceramics-containing composite hydrogels. **a** Expression of dentin-related genes in DPSCs cultured with composite hydrogels for 5 days (*n* = 3). Representative immunofluorescent images of **b** DMP-1 and **c** DSPP proteins in DPSCs after cultured with GelMA and GelMA-5LCS hydrogel for 5 days. (Blue: cell nuclei; Green: cytoskeleton; Red: proteins). **d** Digital images of alizarin red S staining of DPSCs after 10 days of culture. **e** Quantitative results of alizarin red S staining (*n* = 4). *P < 0.05, **P < 0.01, ***P < 0.001. GelMA-5LCS composite hydrogels could significantly enhance the odontogenic differentiation of DPSCs

Live/dead staining and CCK-8 results (Figs. S13 and 5a) confirmed that DPSCs survived well throughout the culture period. GelMA-5LCS group slightly promoted DPSC proliferation, while higher LCS content inhibited it. Moreover, DPSCs also spread well on the surface of these composite hydrogels. However, it is worth noting that the cell density on the composite hydrogel with 10% and 15% LCS concentrations was slightly lower than that in the other groups, corresponding to the CCK-8 results (Fig. 5b). Besides, cell migration activity was evaluated by Transwell assay. As shown in Fig. 5c, a significantly higher number of migrated cells was observed in the GelMA-5LCS group as compared with the GelMA groups. On the contrary, the migrated cell number in GelMA-15LCS was lower than that in GelMA group. Statistical analysis showed that the GelMA-5LCS group has the best performance of inducing cell migration (Fig. 5d). The results showed that the inclusion of 5% LCS bioceramics particles could enhance the proliferation and migration of DPSCs. The odontogenic differentiation activity of DPSCs was assessed by RT-qPCR after 5 days of culture. As demonstrated in Fig. 6a, the expression levels of odontogenic-related genes, including DMP-1, DSPP, OPN, and ALP, were found to be significantly elevated in the GelMA-5LCS group compared to the GelMA group. Furthermore, immunofluorescence images (Fig. 6b, c) and semi-quantitative analysis (Fig. S14) also indicated that GelMA-5LCS composite hydrogel obviously promoted the expression of DMP-1 and DSPP proteins in DPSCs as compared with GelMA hydrogel. In addition, alizarin red S staining experiments were utilized to characterize calcium nodules deposition throughout the odontogenic differentiation of DPSCs. Figure 6d, e show that the addition of 5% LCS bioceramics promoted the formation and deposition of calcium nodules, exhibiting its excellent odontogenesis properties. In summary, the GelMA-5LCS group exhibited optimal performance in promoting the proliferation, migration, and odontogenic differentiation of DPSCs.

### Bioceramics-containing composite hydrogel mediating the coupling of neural regulation of odontogenesis

**Fig. 7:**
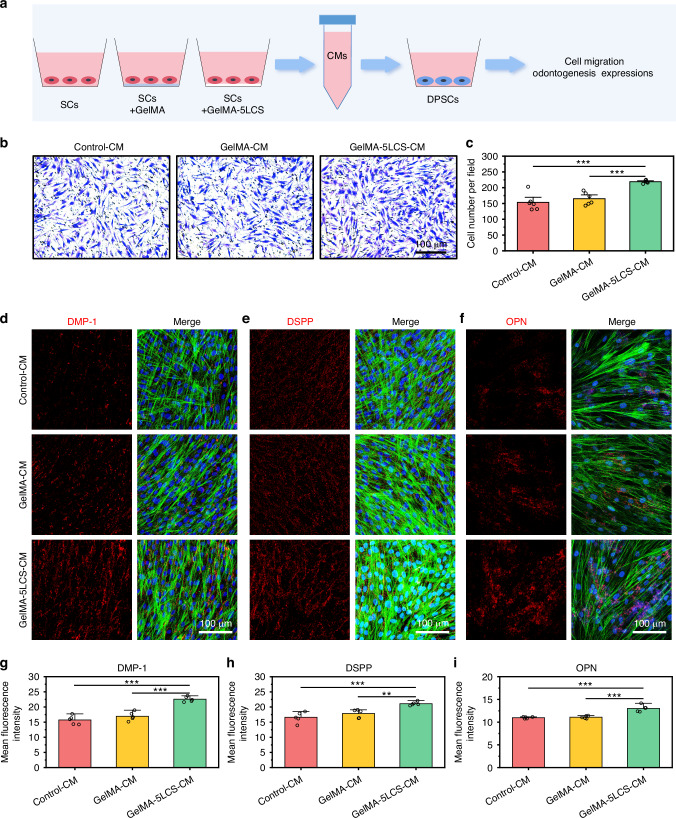
Bioceramics-containing composite hydrogels mediated the neurogenesis- odontogenesis couples. **a** Schematic diagram of the effects of SCs-derived condition medium on the migration and odontogenic differentiation of DPSCs. Conditioned medium was collected from SCs treated with different hydrogels for 2 days, and denoted as Control-CM, GelMA-CM, and GelMA-5LCS-CM, respectively. **b** Representative crystalline violet staining images of DPSCs after cultured with different conditioned medium. **c** Quantitative statistical analysis of the migrated DPSCs (*n* = 6). Representative immunofluorescent staining images of **d** DMP-1, **e** DSPP, and **f** OPN in DPSCs after cultured with different conditioned medium for 5 days (Blue: cell nuclei; Green: cytoskeleton; Red: proteins). Semi-quantitative analysis of **g** DMP-1, **h** DSPP and **i** OPN proteins expression of DPSCs (*n* = 5). *P < 0.05, **P < 0.01, ***P < 0.001. Bioceramics-containing composite hydrogels mediated the neurogenesis-odontogenesis couplings

In this section, we investigated whether the odontogenic differentiation of DPSCs was modulated by neural cells under the treatment of bioceramics-containing composite hydrogels. As shown in Fig. 7a, DPSCs were cultured with conditioned mediums to evaluate the migration and odontogenic differentiation activities. Figure 7b, c showed the crystal violet staining images of migrated cells and its statistical analysis results. It could be observed that the GelMA-5LCS-CM group showed a greater number of migrated cells as compared with the GelMA-CM and Control-CM groups. As demonstrated by immunofluorescence images, the expression levels of odontogenesis-related proteins, including DMP-1, DSPP, and OPN, were found to be significantly elevated in the GelMA-5LCS-CM group in comparison to the other groups (Fig. 7d–f). The results of the semi-quantitative analysis demonstrated that the GelMA-5LCS-CM group exhibited higher expression levels of these proteins in comparison with the Control-CM and GelMA-CM groups (Fig. 7g–i). These results showed that the GelMA-5LCS-CM group had better performance in promoting the migration and odontogenesis of DPSCs as compared with the GelMA-CM group.

### Innervated dentin-pulp regeneration in vivo

**Fig. 8:**
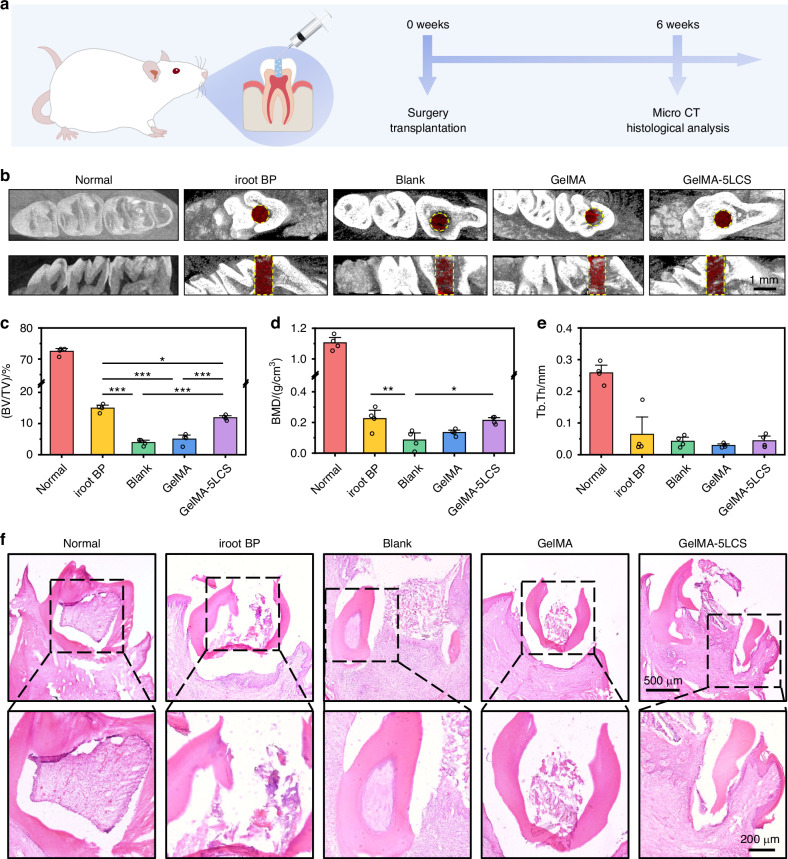
In vivo assay on regeneration of the pulp-dentin complex. **a** Schematic diagram of the animal experiments. **b** Micro-CT reconstruction images in Normal, iRoot BP, Blank, GelMA and GelMA-5LCS groups, respectively. The dotted line delineates the area of the defect, while the red represents the regenerated hard tissue. The statistical analysis results of **c** BV/TV, **d** BMD and **e** Tb. Th (*n* = 4). **f** H&E staining images. *P < 0.05, **P < 0.01, ***P < 0.001. GelMA-5LCS composite hydrogels contribute to the regeneration of dental tissues

In this part, we further investigated the capacity of inducing odontogenesis and neurogenesis in vivo. Dentin-pulp defects with 0.8 mm diameter were created by drilling holes in the molar teeth of rats, as illustrated in Fig. S15. Hydrogels were in situ injected in the defects and crosslinked using blue light. After 6 weeks, jawbone tissues were harvested for further analysis (Fig. 8a).

The collected rat jawbone tissues were scanned by Micro-CT, and the defect site was located (Fig. 8b). Moreover, bone volume/total volume (BV/TV), bone mineral density (BMD), and trabecular thickness (Tb.Th) were obtained by quantitative analysis. As demonstrated in Fig. 8c–e, the GelMA-5LCS group has remarkable higher BV/TV, BMD and Tb.Th values in comparison to the Blank and GelMA groups. Besides, the performance of GelMA-5LCS composite hydrogels was similar with the iRoot BP group, indicating that the incorporation of LCS bioceramics particles obviously enhanced its dentin tissue regeneration capacities.

**Fig. 9:**
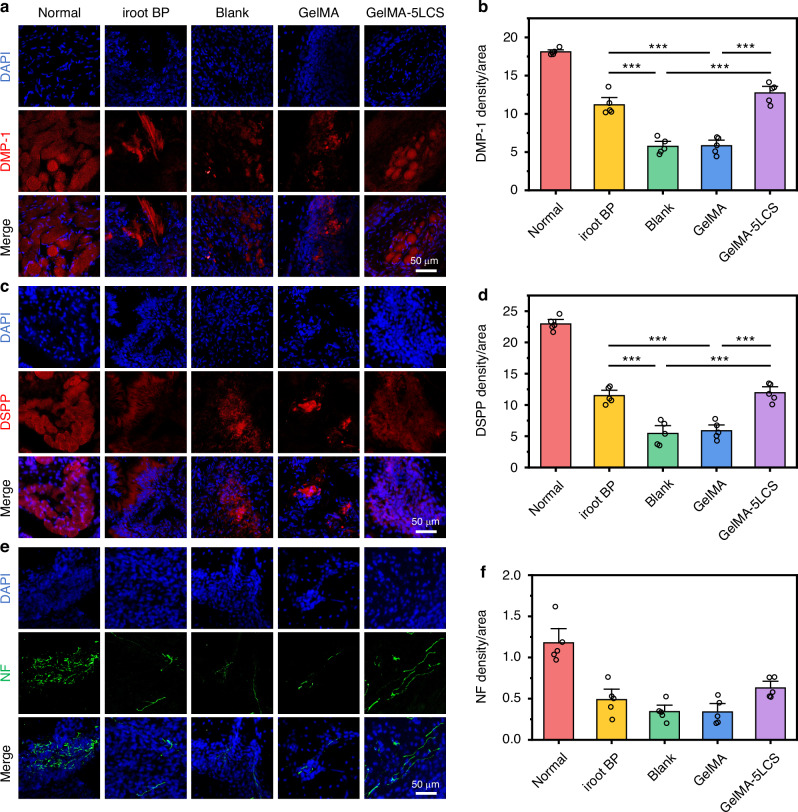
Histological analysis of the repaired pulp-dentin defects after 6 weeks of treatment. **a** Immunofluorescence staining images of DMP-1 in the defect area. **b** Quantitative statistical results of the positive area of DMP-1 (*n* = 5) **c** Immunofluorescence staining images of DSPP in the defect area. **d** Quantitative statistical results of the positive area of DSPP (*n* = 5). **e** Immunofluorescence images of nerve fibers (Neurofilament, NF) in the defects area. **f** Quantitative statistical analysis of the positive area of NF (*n* = 5). *P < 0.05, **P < 0.01, ***P < 0.001. GelMA-5LCS composite hydrogels could promote dentin formation and induce nerve fiber ingrowth, contributing to innervated pulp-dentin complex regeneration

H&E staining results indicated that the pulp tissues in the GelMA-5LCS group were clear and exhibited closer to normal tissue morphology than those in other groups (Fig. 8f). Furthermore, pulp-dentin regeneration and innervation were evaluated by immunofluorescent staining experiments. Dentin markers DMP-1 and DSPP were used to evaluate the dentin formation. As demonstrated in Fig. 9a, c, more DMP-1 and DSPP proteins (red fluorescence) were expressed in GelMA-5LCS and iRoot BP groups as compared with Blank and GelMA groups. And there was no significant difference between the GelMA-5LCS and iRoot BP groups. Semi-quantitative analysis showed that GelMA-5LCS and iRoot BP groups had comparatively large DMP-1 and DSPP positive area, and both were higher than Blank and GelMA groups, which is in accordance with immunofluorescent images (Fig. 9b, d). The ingrowth of nerve fibers was evaluated via immunofluorescence staining of neurofilaments. As demonstrated in Fig. 9e, f, normal tissues have an abundant distribution of nerve fibers, which mediated its physiological functions. More interestingly, compared to Blank and GelMA groups, the GelMA-5LCS group exhibited higher neurofilament density in the defect area, indicating enhanced innervation.

## Discussion

Inspired by the bioactivity of silicate bioceramics, we have prepared an injectable lithium-calcium-silicate (LCS) bioceramics-containing composite hydrogel for innervated pulp-dentin regeneration. The composite hydrogel possesses good injectability, shape fidelity, and photo-crosslinking properties, which is suitable for in situ filling pulp-dentin defects. In comparison with the prolonged curing times of several hours observed for conventional dental products (e.g., MTA and iRoot BP),^49,50^ the composite hydrogel demonstrates a distinctive property of rapid curing, capable of completing photo-crosslinking within a remarkably brief timeframe of 5 to 40 seconds (Figs. 2, 3). Furthermore, as shown in Fig. 2e, composite hydrogels exhibit macro-pore structure, which can support material exchange and cell infiltration, thereby facilitating the provision of a suitable 3D microenvironment for pulp cells and tissues.^51,52^ Therefore, as compared with the current pulp capping agents, which have disadvantages such as inconvenient use, long curing time, and high requirements for clinical operation techniques, the composite hydrogel exhibits suitable convenience in clinical application. Meanwhile, the composite hydrogel has a simpler composition and excellent biocompatibility. The incorporation of bioceramics particles endows hydrogels with enhanced mechanical strength and mineralization ability. In consideration of the augmentation in mechanical strength, a previous study has confirmed that the addition of inorganic biomaterials could enhance the molecular network density of polymer hydrogel, thus affecting its mechanical strength.^51^ Therefore, the underlying mechanism of these phenomena may be attributed to the enhancement of the molecular network density of the hydrogel with the incorporation of LCS particles. The reason for the enhanced mineralization effect is that LCS bioceramics can release Ca²⁺ ions and, after a series of reactions, eventually lead to the precipitation of apatite on the surface of the composite hydrogel material.^53,54^ Moreover, the formation of hydroxyapatite layer can enhance the remineralization of damaged dentin in the process of dentin regeneration, thus accelerating dentin repair.^24^

Furthermore, the in vitro results showed that bioceramics-containing composite hydrogel could promote the neurogenic differentiation of SCs, odontogenic differentiation of DPSCs, as well as neuro-odontogenesis couplings. We speculated that the excellent bioactivities of the composite hydrogels were mainly caused by the incorporation of LCS bioceramics. Ion release results indicated that LCS bioceramics released multiple bioactive ions, creating a beneficial ionic microenvironment for regulating cellular behaviors such as migration and specific differentiation. Li and Si ions released from LCS bioceramics exerted a synergistic effect in promoting SCs proliferation, migration, and neurogenic differentiation.^55,56^ Li ions also have the ability to promote the expression and nuclear localization of β-catenin, leading to SCs differentiation and myelination.^39,40^ Besides, Li ions exhibited a promotion effect on migration, odontogenic differentiation, and mineralization of DPSCs.^41^ In addition, the in vitro experiment also confirmed that GelMA-LCS hydrogels could induce mineralization, which is helpful to dentin formation.^23,57,58^ Among them, GelMA-5LCS has the best neurogenic and odontogenic activities due to the release of various ions within the appropriate concentration range. Numerous studies have found that the excessive ions concentration from bioceramics may induce potential cytotoxicity, which is harmful to cell viabilities.^32,59–62^ While the less ions release also shows limited bioactivity. Bioactive ion release within an appropriate concentration range exhibit optimal biological activities, including enhancing the proliferation, migration, and differentiation behaviors. Therefore, the composite hydrogel is speculated to enhance the neurogenic differentiation of neural cells, helping create a suitable neuro-modulatory microenvironment for odontogenesis. Hence, the bioceramics-containing composite hydrogel is expected to promote dentin-pulp integrated repair through the coupling of neuro-odontogenesis.

The in vivo results showed that bioceramics-containing composite hydrogel could not only promote dentin formation but also induce nerve ingrowth, achieving innervated pulp-dentin regeneration. We speculated that the enhanced dentin formation and innervation were mainly due to the incorporation of LCS bioceramics. Multiple bioactive ions released from the composite hydrogel could create suitable ionic microenvironments for tissue regeneration. On the one hand, released Li, Ca, and Si ions can directly enhance the proliferation, migration, and specific differentiations of both DPSCs and SCs, promoting tissue regeneration. On the other hand, we confirmed that the composite hydrogel provides a more suitable neuro-modulatory microenvironment, inducing early innervation and further enhance dentin regeneration. Additionally, the augmented neurogenic activity suggests that SCs have the capacity to secrete various neurotrophic factors, including GDNF, NGF, BDNF, etc. These neurotrophic factors have been demonstrated to provide nutritional support for nerves and promote axonal regeneration.^6,16^ In addition, they also enhance the expression and mineral deposition of odontogenic-related proteins (e.g., DSPP, ALP, OPN) in dental pulp cells, thereby promoting their odontogenic differentiation and mineralization.^63^ Furthermore, the migration of DPSCs is promoted by extracellular vesicles of SCs through the SDF-1/CXCR4 axis and have the potential to recruit endogenous stem cells for tissue repair.^6,64^ In addition, the extracellular vesicles of SCs contain a variety of proteins necessary for tooth regeneration, including TGF-β, wnt5a, and Col1. These proteins have the capacity to regulate the proliferation, migration, and differentiation of cells, thereby promoting the regeneration of the pulp-dentin complex.^6,16^ Taken together, these results demonstrate that a bioceramics-containing composite hydrogel possesses excellent pulp-dentin regeneration with innervation capacity.

Although bioceramics-containing composite hydrogel has shown satisfactory results in the regeneration of the innervated pulp-dentin complex, further studies need to be carried out to clarify some issues. Firstly, the underlying biological mechanism of nerve fiber growth and SCs regulating the behavior of DPSCs under the stimulation of composite hydrogel needs to be further studied. Secondly, in order to better evaluate the clinical relevance of the composite hydrogel and the need for clinical transformation, further characterizations, including mechanical strength of regenerated dentin, long-term stability and the degradation process of the composite hydrogel in the dental environment, functional recovery of regenerated dentin-pulp complex, and systematic comparison with commercial products are needed to be conducted in animal studies to facilitate a multifaceted assessment of the innervated pulp-dentin complex regeneration. Concurrently, it is known that immunomodulatory and antibacterial properties are of great significance in the evaluation of dental implant materials due to the microbial colonization and excessive inflammatory response of oral microenvironments.^19,65^ The antibacterial and anti-inflammatory properties of composite hydrogel merit further investigation. In addition, the effects and mechanisms of composite hydrogel on other cells related to pulp-dentin complex regeneration, such as immune cells and endothelial cells, are worthy of further investigation.

Overall, our study presented an injectable bioceramics-containing composite hydrogel with both neurogenic and odontogenic bioactivities, representing a promising approach for functional pulp-dentin integrated repair.

## Materials and methods

### Materials

Tetraethyl orthosilicate (TEOS) was sourced from Lingfeng Chemical Reagent Co., Ltd (China). Ca(NO_3_)_2_⋅4H_2_O (AR, 99%) and LiNO_3_ (AR, 99%) were acquired from Aladdin Biochemical Technology Co., Ltd (China). Methacrylic anhydride (MA, 94%) was purchased from Adamas (China). Porcine Gelatin (Type A, ~ 300 g Bloom,) and photoinitiator lithium phenyl (2,4,6-trimethylbenzoyl) phosphinate (LAP) were purchased from Sigma-Aldrich (USA).

### Synthesis and characterization of Li_2_Ca_2_Si_2_O_7_ bioceramics particles

Li_2_Ca_2_Si_2_O_7_ (LCS) particles were synthesized by sol-gel method.^33,47^ Briefly, TEOS, deionized water, and 2 mol/L dilute nitric acid were mixed at the molar ratio of TEOS: H_2_O: HNO_3_ = 1: 8: 0.08. The solution was stirred for 1 h, and subsequently, Ca(NO_3_)_2_⋅4H_2_O and LiNO_3_ were added to the solution in the same molar amounts as TEOS, respectively. After stirring for 6 h, the solution was aged at 60 °C for 24 h and dried at 120 °C for 48 h. The as-formed xerogels were ball milled at 500 r/min for 20 minutes and sieved to 20 mesh to obtain the powders. Then the powders were maintained at 900 °C for 2 h at a heating rate of 2 °C/min. The phase of calcined particles was analyzed using an X-ray diffractometer (XRD, D8 ADVANCE, Bruker Co., Germany). A scanning electron microscope (SEM, SU8220, Hitachi, Japan) was used to observe and photograph the morphology of LCS particles. The size distribution of LCS particles was tested by a laser particle meter (Bettersize2600).

### Synthesis of gelatin methacryloyl hydrogel

Gelatin methacryloyl (GelMA) was prepared through the reaction of gelatin with MA.^45,48^ At first, 20 g gelatin was added to 200 mL deionized water, and stirred at 50 °C for about 30 minutes for complete dissolution. After that, 12 mL MA was added to the gelatin solution and reacted under magnetic stirring in a 50 °C water bath for 1.5 h. The solution was then centrifuged for 3 minutes at 3 500 r/min, and the supernatant was collected and diluted 4 times with deionized water. The diluted solution was packed in a dialysis bag (14 kD) and dialyzed in deionized water at 40 °C for 10 days. Finally, the solution was frozen at -80 °C overnight and lyophilized for 2 days. The dried GelMA was kept sealed at room temperature.

### Preparation and characterization of the injectable bioceramics composite hydrogel

Firstly, LAP was dissolved in PBS solution (0.25 %), then 10 % dried GelMA was added to the solution and dissolved at 55 °C. The as-formed GelMA solution was sterilized using the 0.22 μm bacterial filter (Millipore, USA). In addition, a certain mass of LCS bioceramics particles (0, 0.015, 0.030, and 0.045 g) was irradiated under an ultraviolet lamp for 2 h for sterilization, and subsequently added into the GelMA solution (3 mL) to form composite hydrogel, which was further crosslinked by blue light (EFL-LS-1601, EFL, China) for 20 s. Based on the LCS particle to GelMA matrix mass ratio (0%, 5%, 10% and 15%), the composite hydrogels with different contents of LCS were referred to as GelMA, GelMA-5LCS, GelMA-10LCS, and GelMA-15CS, respectively.

The interior macropore structure of the composite hydrogel was characterized by SEM (SU-8200). Briefly, the hydrogels were deep-frozen at −80 °C overnight and lyophilized before being cut along with the longitudinal directions. The morphology of the vertical section of the hydrogel was observed and photographed by SEM. At the same time, the element distribution of the hydrogel was also obtained by energy dispersive spectroscopy. Besides, the phase of the hydrogel was analyzed by XRD.

A rheometer (MCR301, Anton-Paar, Austria) was used to characterize the rheological properties of the composite hydrogels. The change in storage modulus (G’) and loss modulus (G”) of the composite hydrogels was measured with the variation of strain at 10 °C. In addition, the change in viscosity of the composite hydrogels was characterized as the shear rate varied from 0 s^−1^ to 100 s^−1^. The modulus of the composite hydrogels was tested over a frequency range of 0.1 to 10 Hz. The variation of the viscosity of the composite hydrogels was measured when the temperature was varied from 10–40 °C.

The injectability and plasticity of the composite hydrogel were evaluated by extruding the mixture through a 25-gauge needle to write the appointed word ‘SICCAS’ and injecting the mixture into molds with different shapes. The photo-crosslinking characteristics of the composite hydrogel were examined using the rheometer (MCR301).

The swelling properties of the composite hydrogel were tested according to previous reports.^45^ Firstly, hydrogel blocks were prepared using cylindrical molds with a diameter and height of both 15 mm. The hydrogel blocks were lyophilized with their dry weight (*M*_*d*_) recorded. Then the samples were soaked in PBS for 24 h, gently removed the free water on the surface of the hydrogel, and weighed (*M*_*w*_). Finally, the swelling ratio of the composite hydrogel was calculated according to the equations ${M}_{w}/{M}_{d}$, and the equilibrium water content (EWC) was calculated according to the equations $\left({M}_{w}-{M}_{d}\right)/{M}_{d}\times 100 \%$.

A mechanical tester (Instron 5969, USA) was used to determine the elastic modulus of the composite hydrogels. The hydrogel samples for testing were prepared in cylindrical molds with a diameter and height of 15 mm. The measurements were performed under uniaxial compression with 1 mm/min strain rate. The elastic modulus was calculated from the slope of the stress-strain curve within the 0–10% strain range.

To assess the pH of the composite hydrogel immersion solution, a composite hydrogel disc with 8 mm diameter was soaked in 1 mL PBS solution and placed at 37 °C for 1, 3, 7, 11, 14, and 21 days. The pH of the solution was determined at each time point. Meanwhile, the mass of the hydrogel was measured. The initial mass of the hydrogel was designated as *M*_*1*_, and the mass after soaking was designated as *M*_*2*_. The degradation ratio of the hydrogel was calculated according to the formula $\left({M}_{1}-{M}_{2}\right)/{M}_{1}\times 100 \%$.

### Pulp-dentin defect filling simulation experiment

A molar tooth was provided by Shanghai Ninth People’s Hospital. A dental drill was used to create a hole in the molar teeth to simulate pulp-dentin defects. Subsequently, the composite hydrogel stained with red dye was injected into the molar defects to evaluate its filling properties. After cross-linked with blue light for 5 s, the molar teeth filled with hydrogel were soaked in simulated oral fluid (SOF) and placed on a shaker at 37 °C to observe its physiological stability.

### Mineralization ability evaluation

To evaluate in vitro mineralization ability, bioceramics composite hydrogel discs were soaked in SOF solution in a shaker at 37 °C for 1 and 3 days, followed by lyophilized in a freeze-dryer. Subsequently, the mineral deposition behaviors of composite hydrogels were characterized by XRD and SEM.

### Cell culture

Rat Schwann cells (SCs) and human dental pulp stem cells (DPSCs) were utilized in this study. SCs were provided by the Cell Bank of the Chinese Academy of Sciences, while DPSCs were provided by the Shanghai Ninth People’s Hospital. SCs were maintained in Dulbecco’s modified Eagle’s medium (DMEM, low glucose, Sangon, China) containing 10% fetal bovine serum (FBS, Invitrogen, USA) and 1% penicillin-streptomycin (P/S, Invitrogen, USA). Experiments were conducted using SCs from generations 6–9. DPSCs were maintained in the minimum essential medium-α (MEM-α, Sangon, China) containing 10% FBS and 1% P/S, and experiments were performed with DPSCs from generations 2–5. All cells were maintained in 5% CO_2_ atmosphere at 37 °C.

### Cell survival assay

Live/dead staining was used to evaluate the survival behaviors of SCs and DPSCs. In brief, cells (5 000 per well) were seeded on the hydrogel surface in a 48-well plate and cultured for 1, 3, and 5 days. At each time point, the medium was removed and cells were washed three times with PBS. The cells were then stained with a live/dead staining solution at 37 °C for 20 minutes. The Calcein-AM/PI staining kit (Dojindo, Japan) was prepared in PBS with a volume ratio of 1 000: 2: 3 (PBS: AM: PI). After staining, the cells were photographed using a microscope (DMi8S, Leica Microsystems, Germany), with live cells showing green and dead cells showing red.

### Cell proliferation assay

The effect of the bioceramics composite hydrogel on cell proliferation was investigated by the Cell counting Kit-8 method (CCK-8, Dojindo, Japan). Briefly, cells (5 000 per well) were seeded on the hydrogel surface in a 48-well plate and cultured for 1, 3, and 5 days, with medium changes every two days. To replace the original medium at each time point, 300 μL cell culture medium supplemented with 10% CCK-8 solution was added into each well. The cells were then cultured at 37 °C for 1.5 h. 100 μL supernatant was aspirated from each well and added into a new 96-well plate accordingly, and absorbance was detected at 450 nm using a microplate reader (Spectra Fluor Plus, Tecan, Germany).

### Cell morphology observation

The morphology of cells seeded on the composite hydrogel surface was examined to evaluate its surface cytocompatibility. In brief, cells (20 000 per well) were seeded on the hydrogel surface in a 24-well plate. After culturing for 2 days, 4% paraformaldehyde was used to fix the cells for 1 h, followed by washing with PBS. The cells were then permeabilized with 0.1% Triton-X 100 (Sigma-Aldrich, USA) solution for 5 minutes and washed three times with PBS. The cytoskeleton was stained with ‌FITC (Sigma-Aldrich, USA) for 1 h, and the nuclei were subsequently stained with DAPI (Sigma-Aldrich, USA) for 10 minutes in a dark environment. After staining, the cells were washed three times with PBS. The cell morphology was observed and photographed by a confocal laser scanning microscope (TCS SP8, Leica, Germany).

### Cell migration assay

The effect of the composite hydrogel on cell migration activity was assessed using the Transwell assay. In brief, 10 000 cells per well were seeded into the upper chamber of a 24-well plate and cultured for adherence. After 6 h, the hydrogel was placed in the bottom chamber and the medium was replaced with serum-free medium for another 12 h. Subsequently, cells in the chamber were fixed for 1 h. Unmigrated cells on the top surface of the upper chamber were carefully removed with a cotton-tipped applicator, while migrated cells that were located on the bottom surface were subjected to 0.1% crystal violet staining for 3 minutes. The residual stain was then washed away with PBS solution. Afterwards, the migrated cells were observed and photographed by the microscope (DMi8S, Leica Microsystems, Germany), followed by quantification with Image J software.

### Gene expression analysis

To assess the capacities of bioceramics composite hydrogel to induce neurogenesis and odontogenesis, the expression of odontogenic genes in DPSCs and neurogenic genes in SCs was measured by RT-qPCR. At first, the cells (100 000 per well) were seeded on the hydrogel surface in a 6-well plate and cultured for 5 days. Afterwards, total RNA was extracted by Trizol reagent (Invitrogen, USA), followed by sequential use of trichloromethane (Sigma-Aldrich, USA) and 2-propanol (Sigma-Aldrich, USA) to obtain RNA precipitates. RNA was then reverse transcribed into cDNA by the PrimeScript 1st Strand cDNA Synthesis Kit (TOYOBO, Japan). Finally, StepOnePlus real-time system (Applied Biosystems, USA) was used for gene expression analysis. GAPDH was regarded as the housekeeping gene, and the 2^-ΔΔCt^ method was applied for the expression level calculation of related genes. All primer sequences are listed in Table S2.

### Immunofluorescent protein staining assay

Immunofluorescence staining experiments were applied to study the effect of the composite hydrogel on the specific proteins’ expression levels of DPSCs and SCs. Briefly, 50 000 cells per well were seeded on the surface of the composite hydrogel in a 24-well plate and cultured for 5 days. After that, the cells were fixed with 4% paraformaldehyde for 1 h and subsequently washed with PBS solution. After fixation, the cells were permeabilized using 0.1% Triton-X 100 solution for 5 minutes and then rinsed three times with PBS. Cells were then incubated by primary antibodies overnight at 4 °C after blocking by 5% BSA for 30 minutes at room temperature. The next day, the cells were washed with PBS to remove the residual primary antibody solution. Afterwards, the cells were incubated with secondary antibody (1:1 000) in a dark environment for 1 h at 37 °C. The cytoskeleton was then stained with FITC in the dark for 45 minutes, and followed by staining of nuclei with DAPI for 10 minutes. Fluorescence images were photographed by the confocal microscope (TCS SP8) and analyzed in ImageJ software.

### Alizarin Red S (ARS) staining

To characterize the deposition of calcium nodules during the odontogenic differentiation of DPSCs in vitro, the ARS staining experiment was conducted. At first, the bottom chamber of a 24-well plate was seeded with 10 000 cells per well, while bioceramic composite hydrogels were placed into the upper chamber. After 10 days of cell culture, the DPSCs were fixed with 4% paraformaldehyde for 1 h. Cells were subsequently stained with ARS solution (2%, Beyotime, China) at 37 °C for 30 minutes. After that, the cells were washed with ultrapure water until no floating color remained. The stained cells were observed and photographed by the microscope (DMi8 S, Leica, Germany). To quantify the ARS staining values, the samples were treated with 10% acetic acid and 10% ammonium hydroxide. A microplate reader (Spectra Fluor Plus) was used to detect the absorbance of the solution at 405 nm.

### Ionic release

To assess the release profile of Li, Ca, and Si from the composite hydrogel, the previous medium was collected at the time of fluid exchange, and then the solution was analyzed through inductively coupled plasma (ICP, Varian 715ES, Palo Alto, US). The ion release curves were subsequently calculated based on the results obtained.

### Preparation of conditioned medium

Conditioned medium was used to assess the effects of SCs-treated with composite hydrogel on the odontogenic differentiation of DPSCs. Firstly, SCs (50 000 cells per well) were cultured with composite hydrogel for 2 days, after which the medium was exchanged with serum-free medium. After 24 h, the medium was harvested and centrifuged to remove cell debris. The resulting supernatant was then mixed with MEM-α complete medium in a 1:1 volume ratio to prepare conditioned medium. Among them, SCs cultured on the well plate, pure GelMA hydrogel, and GelMA-5LCS hydrogel were named Control-CM, GelMA-CM, and GelMA-5LCS-CM, respectively. Subsequently, DPSCs were seeded on the 24-well plate (50 000 per well) and placed with conditioned medium for culture. The migration and protein expression of DPSCs were performed as described above.

### Animal studies

Male SD rats (8 weeks, weight at 300–350 g) were used for the animal experiments after approval by the Institutional Animal Care and Use Committee of Shanghai Ninth People’s Hospital Affiliated with Shanghai Jiaotong University, School of Medicine (Ethics number: SH9H-2024-A35-1). The animal modeling methods were referred to in a previous study.^19^ Briefly, dentin-pulp defects with a diameter of 0.8 mm were established by drill and divided into five groups: Normal group, iRoot BP group, Blank group, GelMA group, GelMA-5LCS group. All hydrogels were sterilized and then injected into the defect and crosslinked with blue light for 40 s, and then the cavity was sealed with resin. After 6 weeks, the whole jawbone was collected and fixed for further analysis.

### Micro-CT analysis

Animal samples were scanned using Micro-CT (Skyscan1172, Bruker, Germany) at a resolution of 8.9 μm. Three-dimensional reconstructions were generated with CT-Volume software, and the values of bone volume/total volume (BV/TV), bone mineral density (BMD), and trabecular bone thickness (Tb. Th) were analyzed using the CT-Analyzer program.

### Histological staining

To decalcify hard tissues, all the samples from the in vivo test were soaked in 10% EDTA for 1 month, then immersed in 10% and 30% sucrose solution for dehydration. Afterwards, the tissues were embedded with OCT compound and then cut into 10 μm sections by a freezing microtome (CryoStar NX70, Thermo, USA). For H&E staining, sections were soaked in PBS for 20 minutes to remove residual OCT compound, then stained with H&E staining kit (C0105S, Beyotime, China) according to the instructions. An optical microscope (Zeiss, Germany) was used to photograph the tissue images. For immunohistochemical staining assay, the sections were immersed in PBS solution and then immersed in Proteinase K working solution (P78893, Abcone, China) for antigen recovery. Subsequently, the sections were blocked with PBS containing 10% donkey serum (BL939A, Biosharp, China) and 0.3% Triton-X 100 for 1 h. Following this, the sections were incubated with primary antibody overnight at 4 °C, then with secondary antibody for 1 h at room temperature in a dark environment. The sections were covered with fluorescent mounting medium with DAPI (P0131, Beyotime, China). Immunofluorescence images were captured using the confocal microscope (TCS SP8) and statistically analyzed in ImageJ software.

### Statistical analysis

All data were presented as mean ± standard deviation with n ≥ 3 and analyzed using one-way ANOVA in Origin 2021 software (Origin Laboratories, USA). When *P < 0.05, **P < 0.01, or ***P < 0.001, the difference is considered statistically significant.

## Supplementary information

Supporting Information

## Data Availability

The data that support the findings of this study are available from the corresponding author upon reasonable request.

## References

[1] Chang, B., Ahuja, N., Ma, C. & Liu, X. Injectable scaffolds: Preparation and application in dental and craniofacial regeneration. *Mater. Sci. Eng. R. Rep.* **111**, 1–26 (2017). 28649171 10.1016/j.mser.2016.11.001 PMC5478172

[2] Chen, H. et al. Regeneration of pulpo-dentinal-like complex by a group of unique multipotent CD24a^+^ stem cells. *Sci. Adv.* **6**, 1514 (2020). 10.1126/sciadv.aay1514 PMC7141825 32284993

[3] Galler, K. M., Weber, M., Korkmaz, Y., Widbiller, M. & Feuerer, M. Inflammatory response mechanisms of the Dentine-pulp complex and the periapical tissues. *Int J. Mol. Sci.* **22**, 1480 (2021). 33540711 10.3390/ijms22031480 PMC7867227

[4] Nakashima, M. Bone morphogenetic proteins in dentin regeneration for potential use in endodontic therapy. *Cytokine Growth Factor Rev.* **16**, 369–376 (2005). 15878301 10.1016/j.cytogfr.2005.02.011

[5] Siddiqui, Z. et al. Cells and material-based strategies for regenerative endodontics. *Bioact. Mater.* **14**, 234–249 (2022). 35310358 10.1016/j.bioactmat.2021.11.015 PMC8897646

[6] Wang, D. et al. Schwann cell-derived EVs facilitate dental pulp regeneration through endogenous stem cell recruitment via SDF-1/CXCR4 axis. *Acta Biomater.* **140**, 610–624 (2022). 34852303 10.1016/j.actbio.2021.11.039

[7] Wei, Y. et al. Neural regeneration in regenerative endodontic treatment: an overview and current trends. *Int J. Mol. Sci.* **23**, 15492 (2022). 36555133 10.3390/ijms232415492 PMC9779866

[8] Zheng, L. et al. Injectable decellularized dental pulp matrix-functionalized hydrogel microspheres for endodontic regeneration. *Acta Biomater.* **156**, 37–48 (2023). 36455855 10.1016/j.actbio.2022.11.047

[9] Orti, V. et al. Pulp regeneration concepts for nonvital teeth: from tissue engineering to clinical approaches. *Tissue Eng. Part B Rev.* **24**, 419–442 (2018). 29724156 10.1089/ten.TEB.2018.0073

[10] Liang, C. et al. Bone morphogenetic protein 7 mediates stem cells migration and angiogenesis: therapeutic potential for endogenous pulp regeneration. *Int J. Oral. Sci.* **14**, 38 (2022). 35858911 10.1038/s41368-022-00188-y PMC9300630

[11] Qian, Y. et al. DLP printed hDPSC-loaded GelMA microsphere regenerates dental pulp and repairs spinal cord. *Biomaterials* **299**, 122137 (2023). 37172537 10.1016/j.biomaterials.2023.122137

[12] Walton, R. E. & Ramachandran Nair, P. N. Neural elements in dental pulp and dentin. *Oral. Surg. Oral. Med. Oral. Pathol. Oral. Radio. Endod.* **80**, 710–719 (1995). 10.1016/s1079-2104(05)80256-2 8680980

[13] Hashemi-Beni, B., Khoroushi, M., Foroughi, M. R., Karbasi, S. & Khademi, A. A. Tissue engineering: Dentin - pulp complex regeneration approaches (A review). *Tissue Cell* **49**, 552–564 (2017). 28764928 10.1016/j.tice.2017.07.002

[14] Xu, Y. et al. Inferior alveolar nerve transection disturbs innate immune responses and bone healing after tooth extraction. *Ann. N. Y Acad. Sci.* **1448**, 52–64 (2019). 31095746 10.1111/nyas.14120

[15] Liu, A. Q. et al. Sensory nerve-deficient microenvironment impairs tooth homeostasis by inducing apoptosis of dental pulp stem cells. *Cell Prolif.* **53**, e12803 (2020). 32246537 10.1111/cpr.12803 PMC7260073

[16] Li, Z. et al. Schwann cells secrete extracellular vesicles to promote and maintain the proliferation and multipotency of hDPCs. *Cell Prolif.* **50**, e12353 (2017). 28714175 10.1111/cpr.12353 PMC6529079

[17] Frostick, S. P., Yin, Q. & Kemp, G. J. Schwann cells, neurotrophic factors, and peripheral nerve regeneration. *Microsurgery* **18**, 397–405 (1998). 9880154 10.1002/(sici)1098-2752(1998)18:7<397::aid-micr2>3.0.co;2-f

[18] Liang, Z. et al. Multifunctional Lithium-doped mesoporous nanoparticles for effective dentin regeneration in vivo. *Int J. Nanomed.* **18**, 5309–5325 (2023). 10.2147/IJN.S424930 PMC10516199 37746049

[19] Qiu, Y. et al. SrCuSi_4_O_10_/GelMA composite hydrogel-mediated vital pulp therapy: integrating antibacterial property and enhanced pulp regeneration activity. *Adv. Health Mater.* **12**, e2300546 (2023). 10.1002/adhm.202300546 PMC11469286 37260366

[20] Cooper, P. R. et al. Inflammation–regeneration interplay in the dentine–pulp complex. *J. Dent.* **38**, 687–697 (2010). 20580768 10.1016/j.jdent.2010.05.016

[21] Neves, V. C. M., Yianni, V. & Sharpe, P. T. Macrophage modulation of dental pulp stem cell activity during tertiary dentinogenesis. *Sci. Rep.* **10**, 20216 (2020). 33214653 10.1038/s41598-020-77161-4 PMC7678850

[22] Hilkens, P. et al. The angiogenic potential of DPSCs and SCAPs in an In vivo model of dental pulp regeneration. *Stem Cells Int* **2017**, 2582080 (2017). 29018483 10.1155/2017/2582080 PMC5605798

[23] Wen, B. et al. Biomineralization-inspired mineralized hydrogel promotes the repair and regeneration of dentin/bone hard tissue. *NPJ Regen. Med.* **8**, 11 (2023). 36841873 10.1038/s41536-023-00286-3 PMC9968336

[24] Jin, W. et al. Biomimetic mineralized collagen scaffolds enhancing odontogenic differentiation of hDPSCs and dentin regeneration through modulating mechanical microenvironment. *Chem. Eng. J.* **460**, 141800 (2023).

[25] Duan, Y., Liang, Y., Yang, F. & Ma, Y. Neural Regulations in tooth development and Tooth-Periodontium Complex Homeostasis: A literature review. *Int J. Mol. Sci.* **23**, 14150 (2022). 36430624 10.3390/ijms232214150 PMC9698398

[26] Gao, C. et al. Current progress in bioactive ceramic scaffolds for bone repair and regeneration. *Int J. Mol. Sci.* **15**, 4714–4732 (2014). 24646912 10.3390/ijms15034714 PMC3975421

[27] Wenping, M. et al. Hierarchically structured biomaterials for tissue regeneration. *Microstructures* **4**, 2024014 (2024).

[28] Gou, Y., Qi, K., Wei, Y., Gu, Z. & Xie, H. Advances of calcium phosphate nanoceramics for the osteoinductive potential and mechanistic pathways in maxillofacial bone defect repair. *Nano TransMed* **3**, 100033 (2024).

[29] Yang, Z. et al. 3D printing of sponge spicules-inspired flexible bioceramic-based scaffolds. *Biofabrication* **14**, 035009 (2022). 10.1088/1758-5090/ac66ff 35417888

[30] Du, L. et al. Multicellular Bioprinting of Biomimetic Inks for Tendon-to-Bone Regeneration. *Adv. Sci.* **10**, e2301309 (2023). 10.1002/advs.202301309 PMC10375072 37119499

[31] Zheng, Y. et al. An ultralong hydroxyapatite nanowire aerogel for rapid hemostasis and wound healing. *Chem. Eng. J.* **430**, 132912 (2022).

[32] Zhang, H. et al. Bioprinting of inorganic-biomaterial/neural-stem-cell constructs for multiple tissue regeneration and functional recovery. *Natl. Sci. Rev.* **11**, nwae035 (2024). 38463933 10.1093/nsr/nwae035 PMC10924618

[33] Chen, L. et al. 3D printing of a lithium-calcium-silicate crystal bioscaffold with dual bioactivities for osteochondral interface reconstruction. *Biomaterials* **196**, 138–150 (2019). 29643002 10.1016/j.biomaterials.2018.04.005

[34] Du, L., Xue, J., Huan, Z. & Wu, C. Regulation of Tendon-bone related multiple cells by chemical ions released from Mo-containing Silicate Bioceramics. *Acta Chim. Sin.* **81**, 1334–1340 (2023).

[35] Qin, C. et al. 3D bioprinting of multicellular scaffolds for osteochondral regeneration. *Mater. Today* **49**, 68–84 (2021).

[36] Yang, M. et al. Renal-friendly Li plus -doped carbonized polymer dots activate Schwann cell autophagy for promoting peripheral nerve regeneration. *Acta Biomater.* **159**, 353–366 (2023). 36669552 10.1016/j.actbio.2023.01.027

[37] Farmani, A. R. et al. Li-doped bioactive ceramics: promising biomaterials for tissue engineering and regenerative medicine. *J. Funct. Biomater.* **13**, 162 (2022). 36278631 10.3390/jfb13040162 PMC9589997

[38] Rybakowski, J. K., Suwalska, A. & Hajek, T. Clinical perspectives of lithium’s neuroprotective effect. *Pharmacopsychiatry* **51**, 194–199 (2018). 29270949 10.1055/s-0043-124436

[39] Kuffler, D. P. Can lithium enhance the extent of axon regeneration and neurological recovery following peripheral nerve trauma?. *Neural Regen. Res* **17**, 948–952 (2022). 34558506 10.4103/1673-5374.324830 PMC8552832

[40] Makoukji, J. et al. Lithium enhances remyelination of peripheral nerves. *Proc. Natl. Acad. Sci. USA* **109**, 3973–3978 (2012). 22355115 10.1073/pnas.1121367109 PMC3309729

[41] Ali, M. et al. Lithium-containing surface pre-reacted glass fillers enhance hDPSC functions and induce reparative dentin formation in a rat pulp capping model through activation of Wnt/β-catenin signaling. *Acta Biomater.* **96**, 594–604 (2019). 31212112 10.1016/j.actbio.2019.06.016

[42] Wang, J. et al. Progress on biomimetic mineralization and materials for hard tissue regeneration. *ACS Biomater. Sci. Eng.* **9**, 1757–1773 (2023). 34870411 10.1021/acsbiomaterials.1c01070

[43] Hao, M. et al. Hydroxyapatite nanorods function as safe and effective growth factors regulating neural differentiation and neuron development. *Adv. Mater.* **33**, 2100895 (2021). 10.1002/adma.202100895 34247433

[44] He, J. et al. Synergistic effect of endocellular calcium ion release and nanotopograpy of one-dimensional hydroxyapatite nanomaterials for accelerating neural stem cell differentiation. *Compos Part B-Eng.* **219**, 108944 (2021).

[45] Zhang, H. et al. Calcium silicate nanowires-containing multicellular bioinks for 3D bioprinting of neural-bone constructs. *Nano Today* **46**, 101584 (2022).

[46] Ma, Y.-X. et al. Silicified collagen scaffold induces semaphorin 3A secretion by sensory nerves to improve in-situ bone regeneration. *Bioact. Mater.* **9**, 475–490 (2022). 34820584 10.1016/j.bioactmat.2021.07.016 PMC8586786

[47] Deng, C. et al. Bioactive scaffolds with Li and Si ions-synergistic effects for osteochondral defects regeneration. *Appl. Mater. Today* **10**, 203–216 (2018).

[48] Ouyang, L., Armstrong, J. P. K., Chen, Q., Lin, Y. & Stevens, M. M. Void-Free 3D bioprinting for in situ endothelialization and microfluidic perfusion. *Adv. Funct. Mater.* **30**, 1908349 (2020). 33071714 10.1002/adfm.201908349 PMC7116187

[49] Song, W., Sun, W., Chen, L. & Yuan, Z. In vivo biocompatibility and bioactivity of calcium silicate-based bioceramics in endodontics. *Front Bioeng. Biotechnol.* **8**, 580954 (2020). 33195142 10.3389/fbioe.2020.580954 PMC7658386

[50] Lin, Y. T. et al. The development of light-curable calcium-silicate-containing composites used in odontogenic regeneration. *Polymers* **13**, 3107 (2020). 10.3390/polym13183107 PMC8468725 34578012

[51] He, J. et al. Gelatin methacryloyl hydrogel, from standardization, performance, to biomedical application. *Adv. Health Mater.* **12**, e2300395 (2023). 10.1002/adhm.202300395 37115708

[52] Atila, D. T. et al. In vitro evaluation of injectable Tideglusib-loaded hyaluronic acid hydrogels incorporated with Rg1-loaded chitosan microspheres for vital pulp regeneration. *Carbohydr. Polym.* **278**, 118976 (2022). 34973790 10.1016/j.carbpol.2021.118976

[53] Liu, X. Y., Ding, C. X. & Chu, P. K. Mechanism of apatite formation on wollastonite coatings in simulated body fluids. *Biomaterials* **25**, 1755–1761 (2004). 14738838 10.1016/j.biomaterials.2003.08.024

[54] Wu, C. T. & Chang, J. Silicate bioceramics for bone tissue regeneration. *J. Inorg. Mater.* **28**, 29–39 (2013).

[55] Wei, R., Zhang, Z., Xing, M., Zhou, Y. & Chang, J. Preparation and in vitro evaluation of Lithium-doped bioactive glasses for wound healing with nerve repair potential. *Mater. Lett.* **292**, 129629 (2021).

[56] Gu, X.-K., Li, X.-R., Lu, M.-L. & Xu, H. Lithium promotes proliferation and suppresses migration of Schwann cells. *Neural Regen. Res* **15**, 1955–1961 (2020). 32246645 10.4103/1673-5374.280324 PMC7513976

[57] Niu, L. N. et al. Biomimetic remineralization of dentin. *Dent. Mater.* **30**, 77–96 (2014). 23927881 10.1016/j.dental.2013.07.013 PMC3867526

[58] Zhang, W. et al. The performance of human dental pulp stem cells on different three-dimensional scaffold materials. *Biomaterials* **27**, 5658–5668 (2006). 16916542 10.1016/j.biomaterials.2006.07.013

[59] Zhang, Q. et al. Lithium-calcium-silicate bioceramics stimulating cementogenic/osteogenic differentiation of periodontal ligament cells and periodontal regeneration. *Appl. Mater. Today* **16**, 375–387 (2019).

[60] Qin, C. et al. Cell-laden scaffolds for vascular-innervated bone regeneration. *Adv. Health Mater.* **12**, e2201923 (2023). 10.1002/adhm.202201923 36748277

[61] Ma, W. et al. A bioactive calcium silicate nanowire-containing hydrogel for organoid formation and functionalization. *Mater. Horiz.* **11**, 2957–2973 (2024). 38586926 10.1039/d4mh00228h

[62] Huang, J. et al. An injectable hyaluronic acid/lithium calcium silicate soft tissue filler with vascularization and collagen regeneration. *Bioact. Mater.* **44**, 256–268 (2024). 39507373 10.1016/j.bioactmat.2024.10.014 PMC11539074

[63] Mizuno, N. et al. Effect of neurotrophins on differentiation, calcification and proliferation in cultures of human pulp cells. *Cell Biol. Int* **31**, 1462–1469 (2007). 17720554 10.1016/j.cellbi.2007.06.012

[64] Eramo, S. et al. Dental pulp regeneration via cell homing. *Int Endod. J.* **51**, 405–419 (2018). 29047120 10.1111/iej.12868

[65] Min, Q. et al. Immunomodulatory mechanism and potential application of dental pulp-derived stem cells in immune-mediated diseases. *Int J. Mol. Sci.* **24**, 8068 (2023). 37175774 10.3390/ijms24098068 PMC10178746

