# Supplementary material for: An injectable bioceramics-containing composite hydrogel promoting innervation for pulp-dentin complex repair
Source: Int J Oral Sci. 2025 Oct 1;17:66. doi: 10.1038/s41368-025-00398-0 (PMC12485191; doi:10.1038/s41368-025-00398-0)
Supplement: Supplementary file 1 — Supporting Information [file 41368_2025_398_MOESM1_ESM.docx]

**Supporting Information**


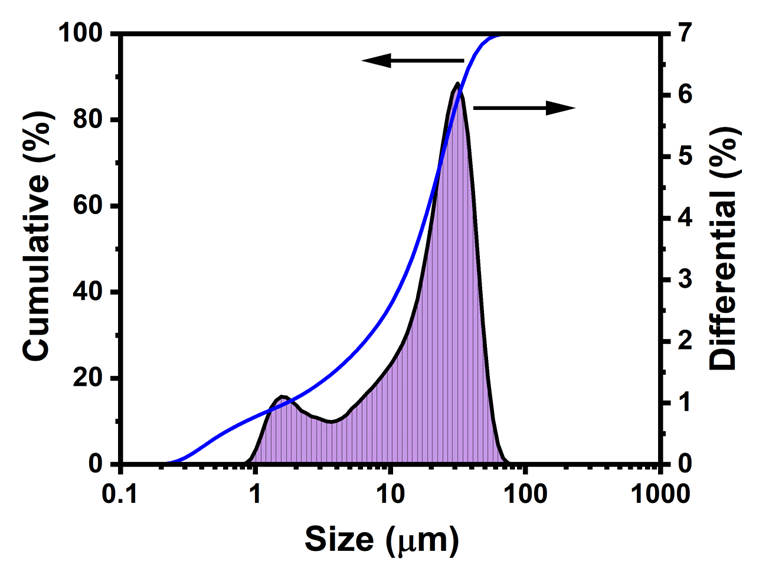


**Fig. S1** The size distribution of LCS particles.


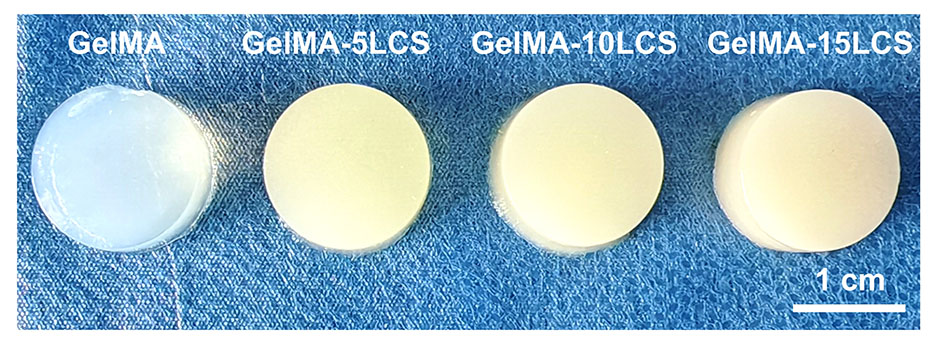


**Fig. S2** Optical images of composite hydrogels with different concentrations of LCS particles.


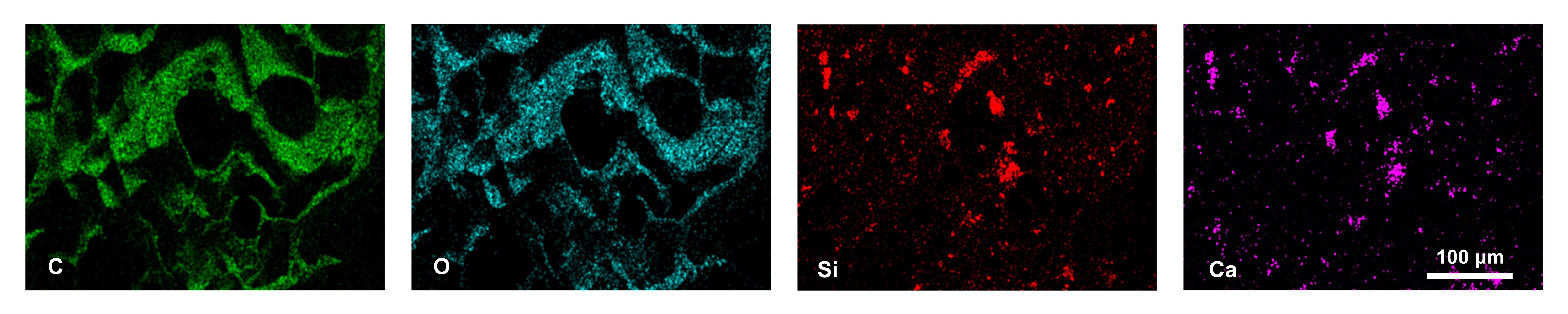


**Fig. S3** The EDS element mapping of GelMA-10LCS hydrogel indicates that LCS particles were uniformly distributed in GelMA hydrogel.


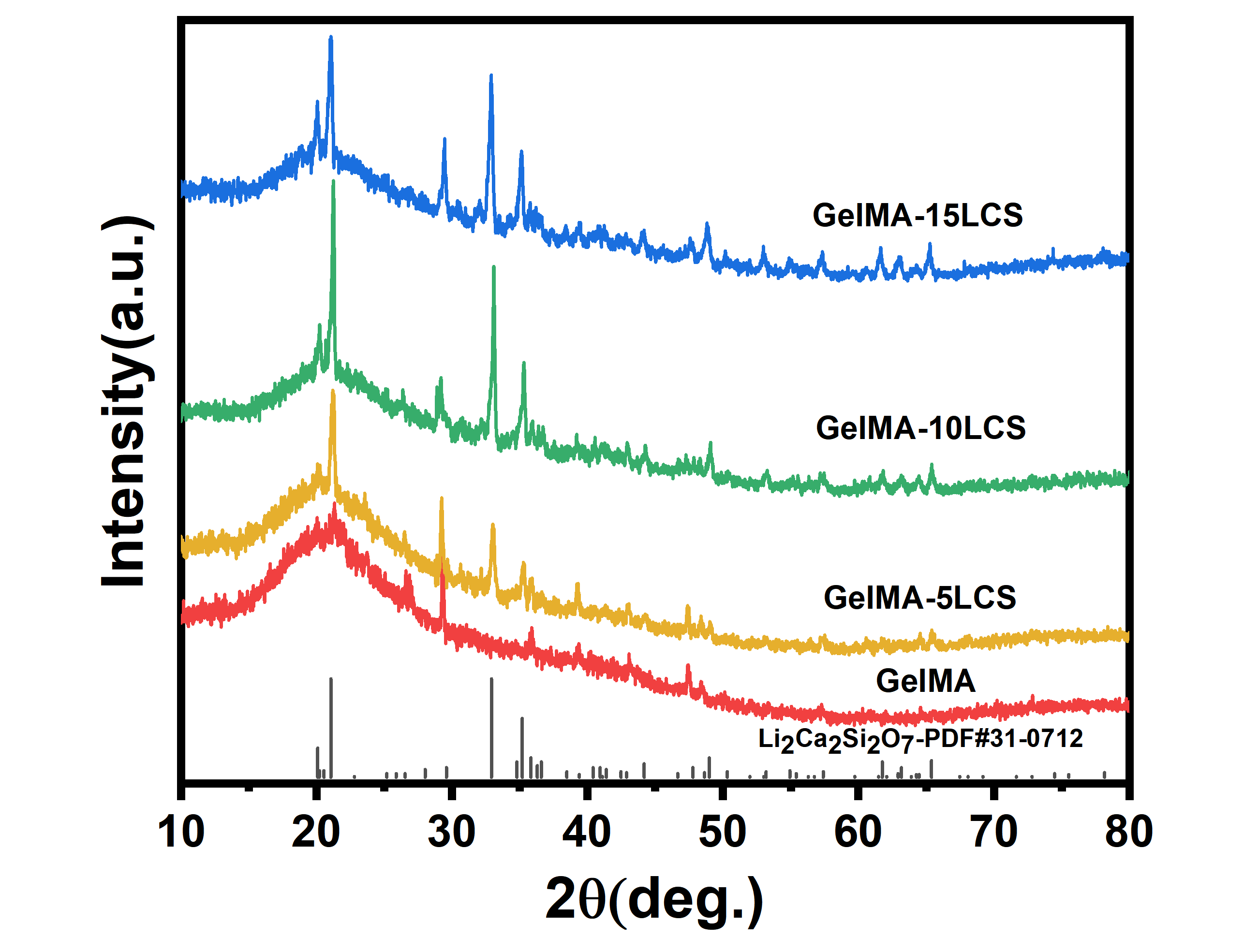


**Fig. S4** XRD pattern of GelMA, GelMA-5LCS, GelMA-10LCS and GelMA-15LCS hydrogels. The LCS bioceramic particles were successfully loaded into the GelMA hydrogel matrix.


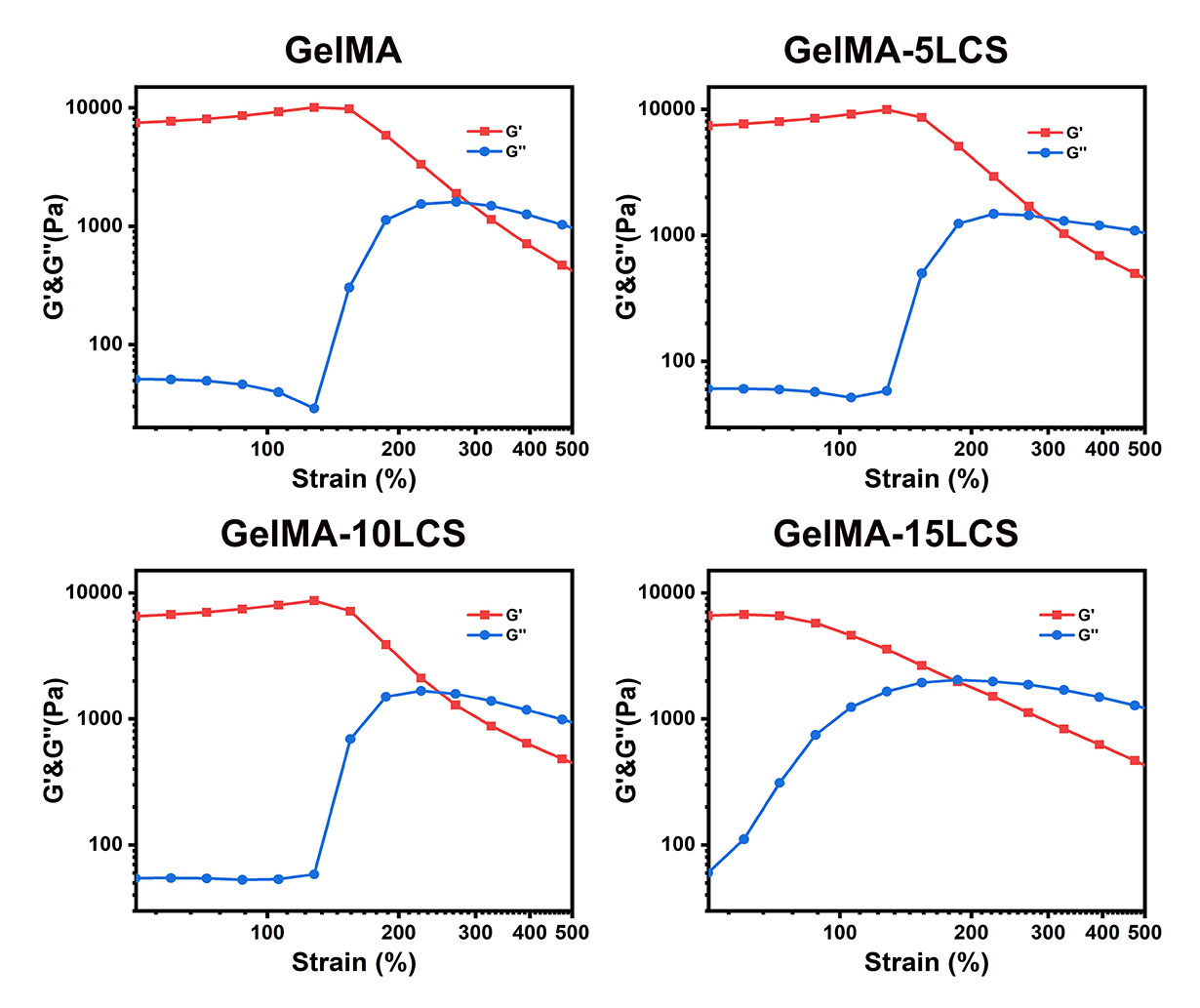


**Fig. S5** The modulus-strain curves of composite hydrogels.


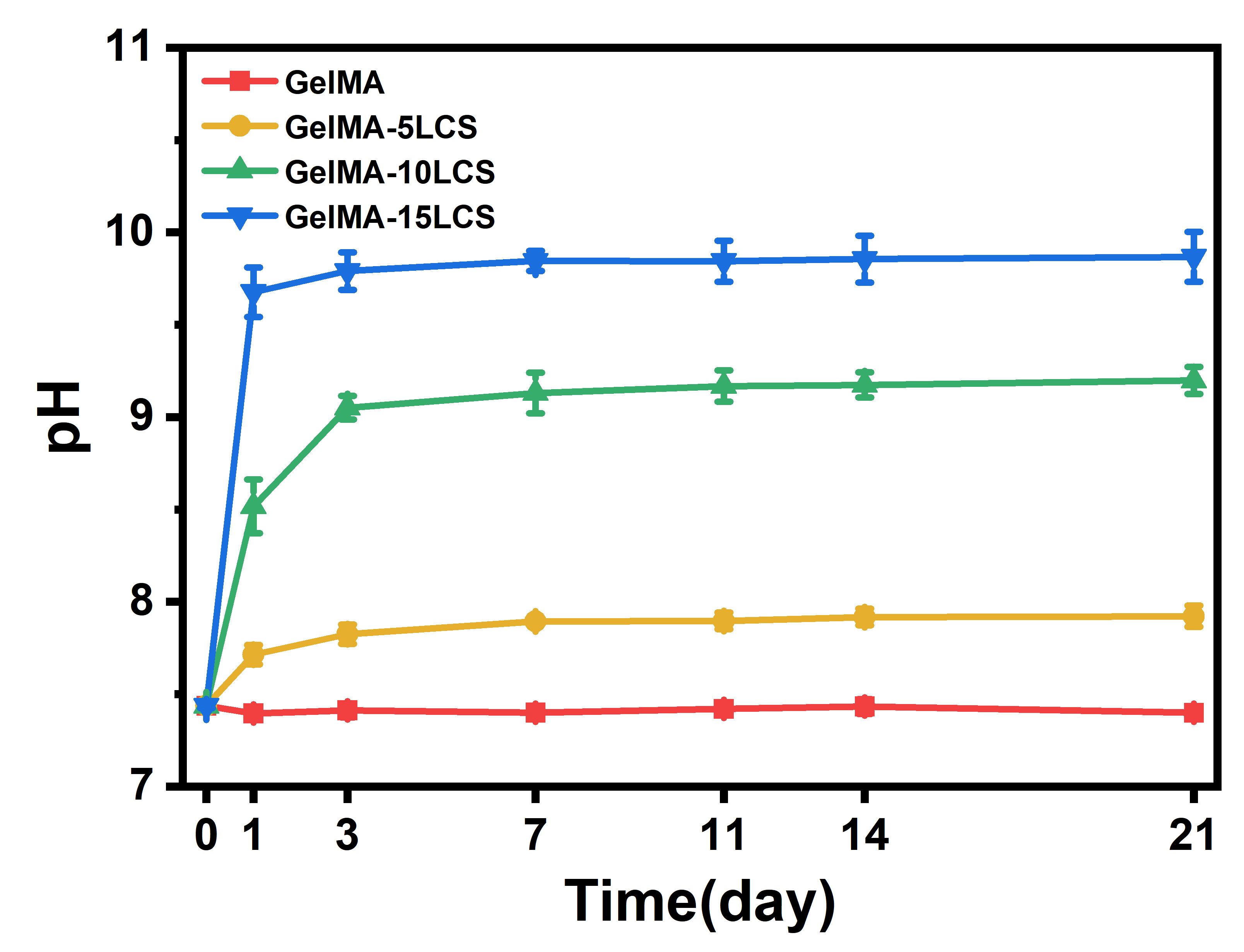


**Fig. S6** The pH values of composite hydrogels immersed in PBS at different periods (*n* = 3). The alkalinity of the solution increased with the increase of LCS bioceramic contents.


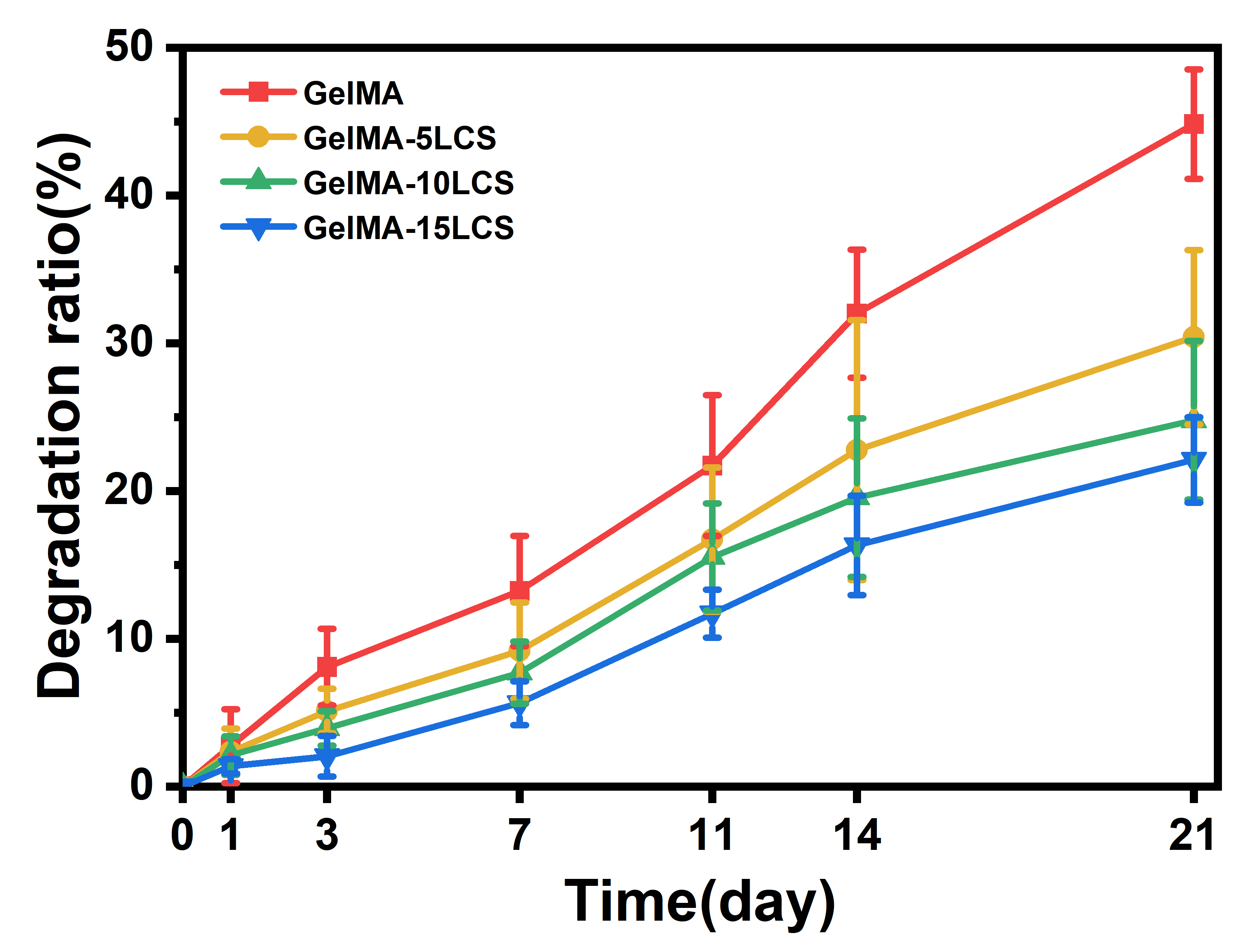


**Fig. S7** Degradation curve of composite hydrogels for 21 days (*n* = 3).


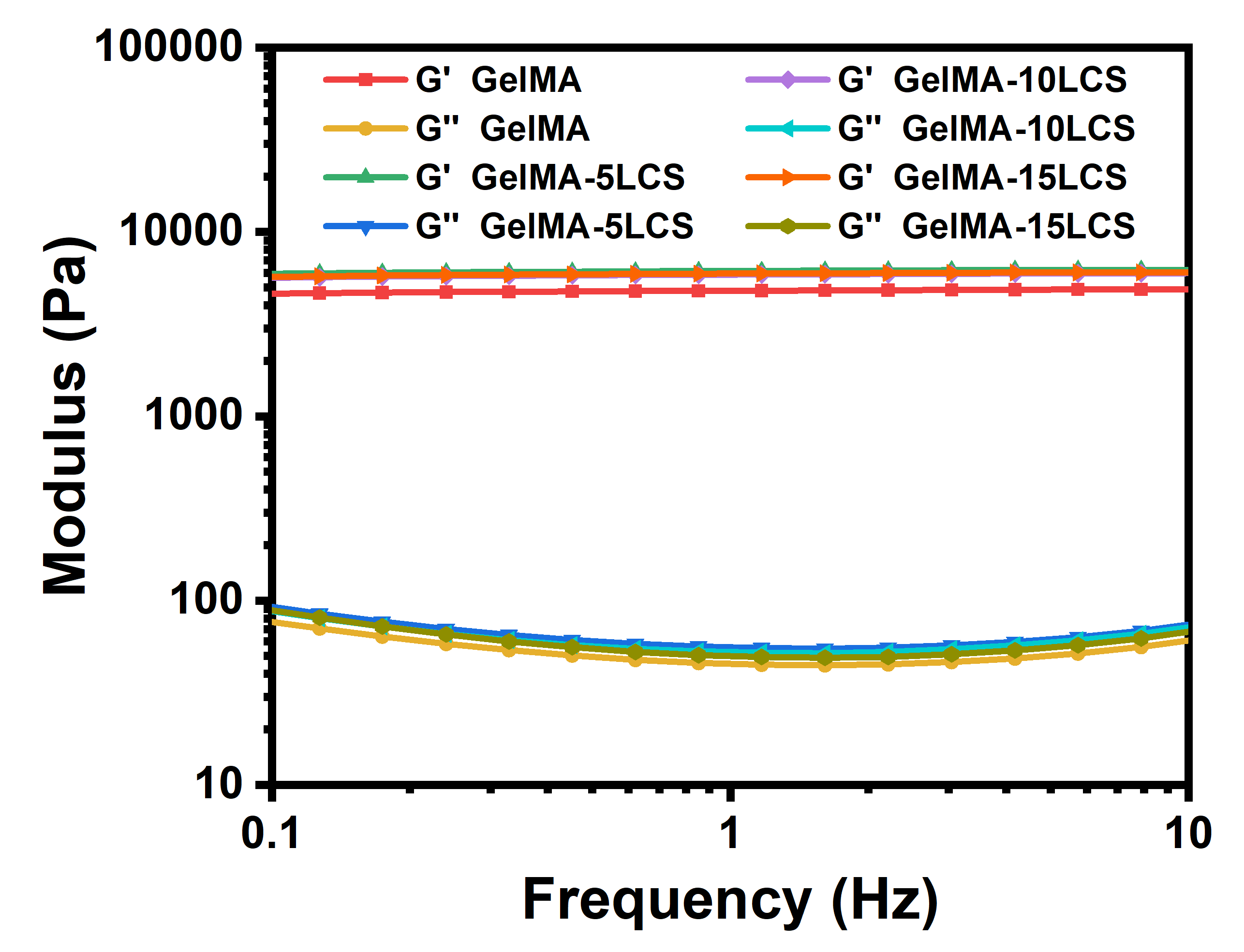


**Fig. S8** The modulus-frequency curves of composite hydrogels.


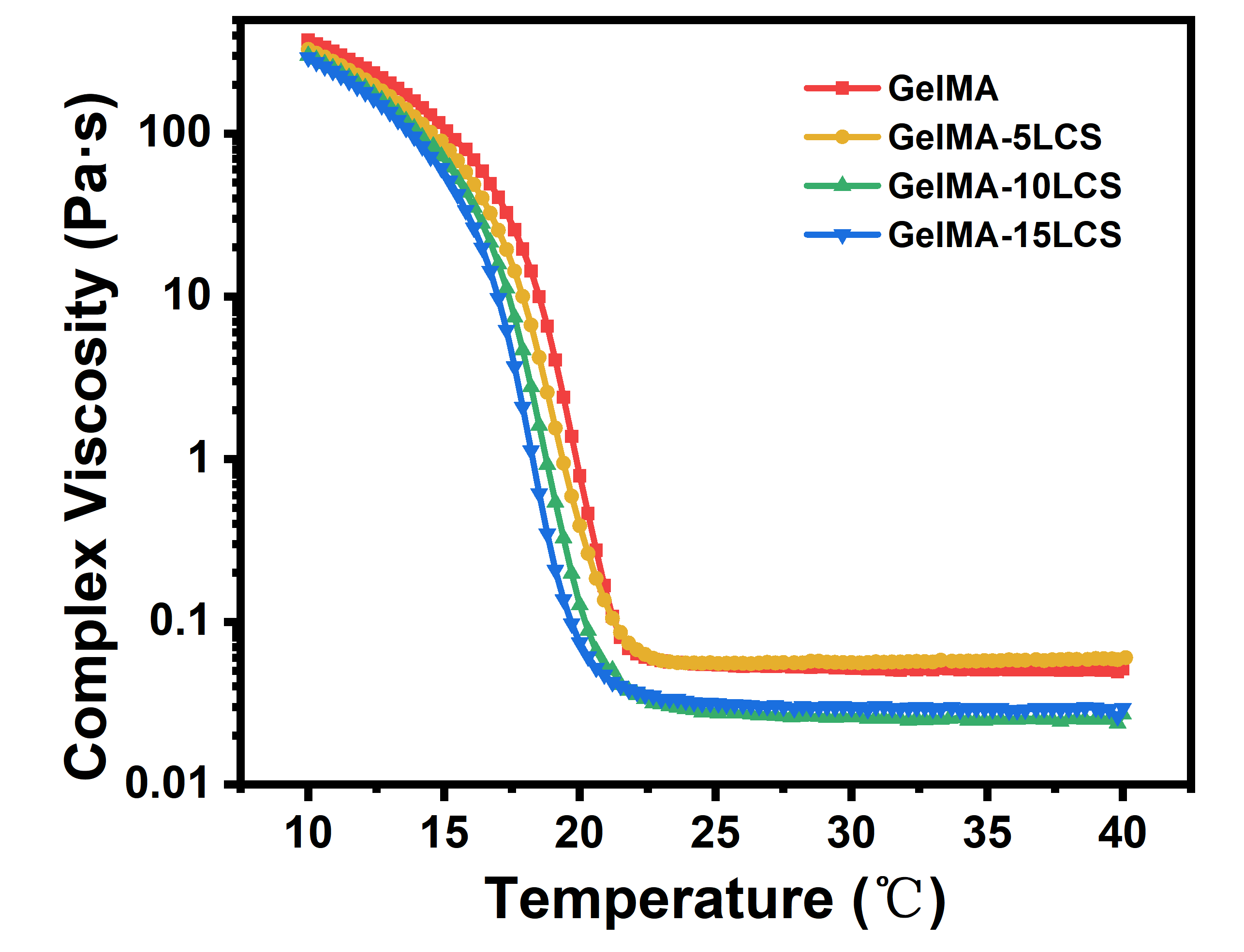


**Fig. S9** The viscosity-temperature curves of composite hydrogels, indicating that the addition of LCS ceramic particles has no significant effects on the temperature sensitivity of GelMA hydrogels.


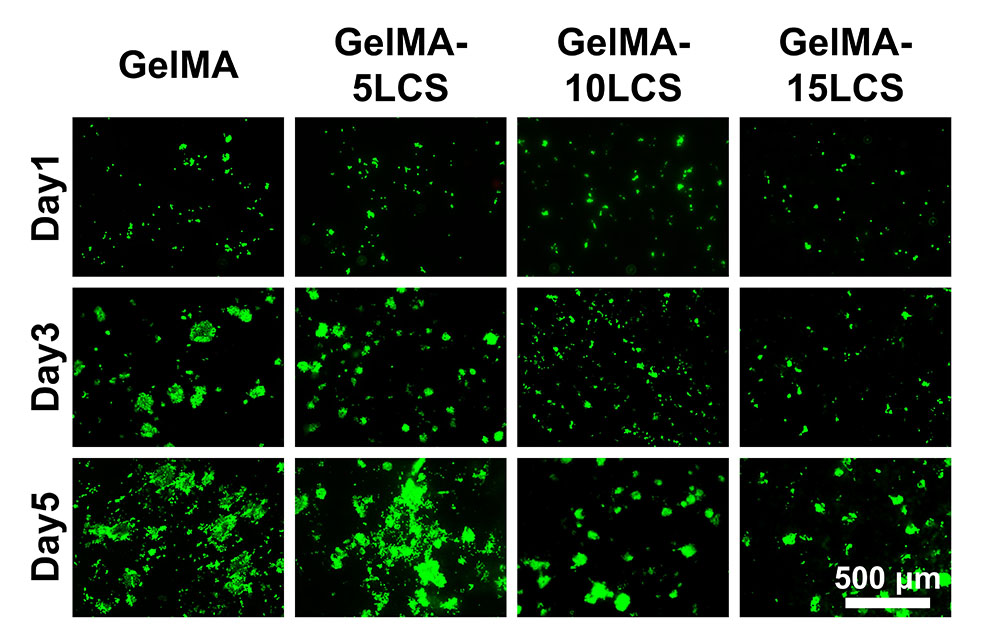


**Fig. S10** Live/dead staining images of Schwann cells (SCs) cultured with different composite hydrogels for 1, 3 and 5 days (Green: live cells; Red: dead cells).


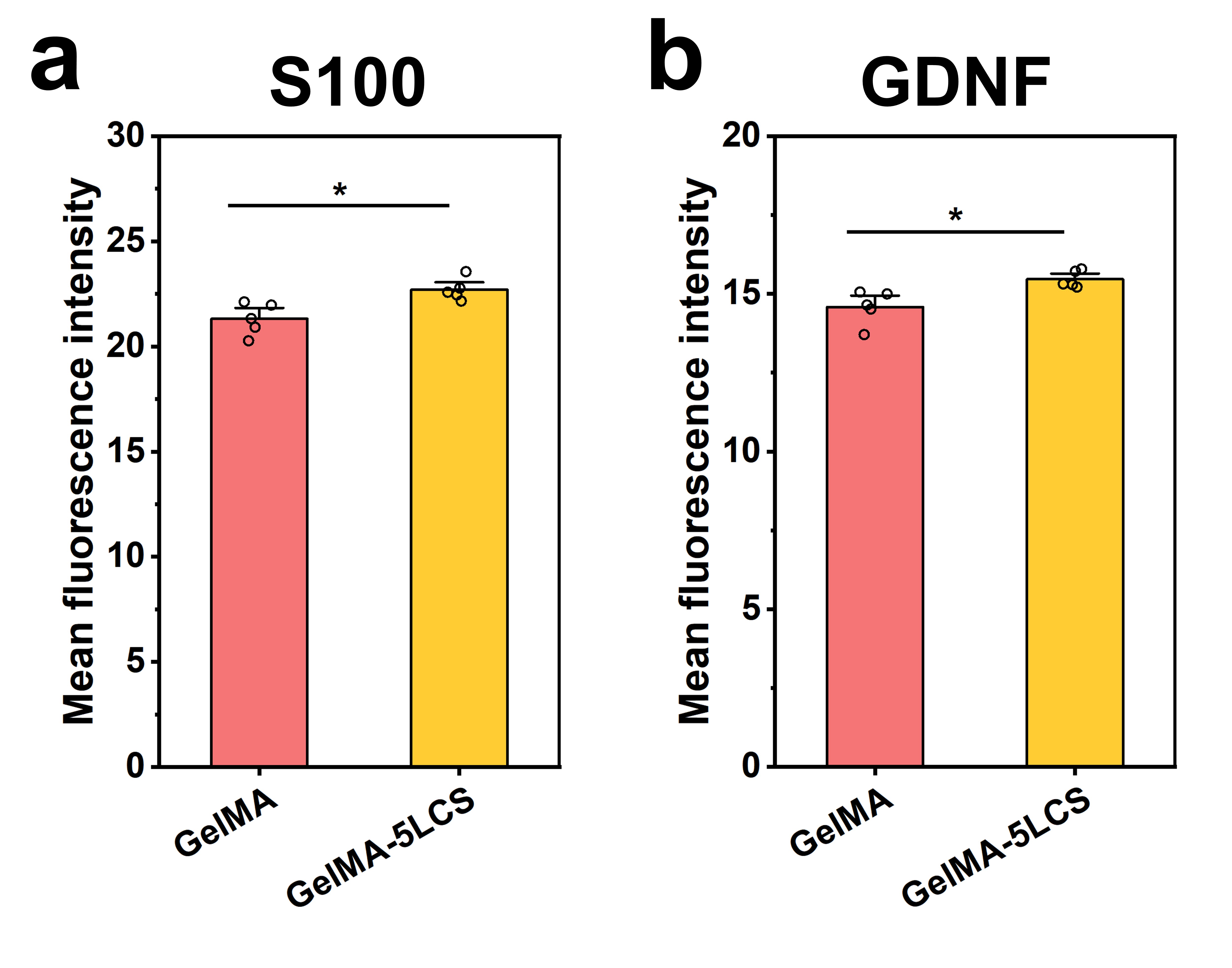


**Fig. S11** Semi-quantitative analysis of **a** S100 and **b** GDNF proteins in SCs after cultured with GelMA and GelMA-5LCS hydrogel for 5 days (*n* = 5).


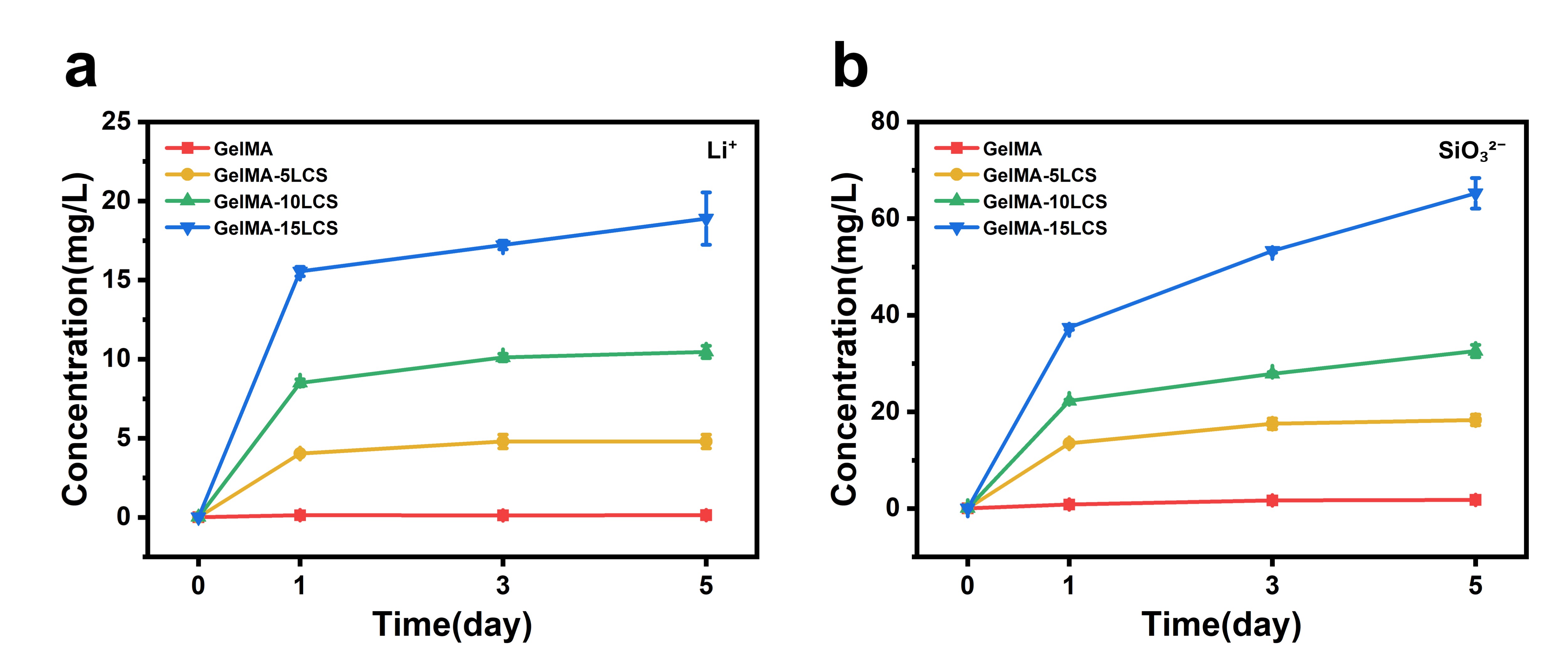


**Fig. S12** The cumulative release curves of **a** Li⁺ and **b** SiO₃²⁻ over time (*n* = 3).


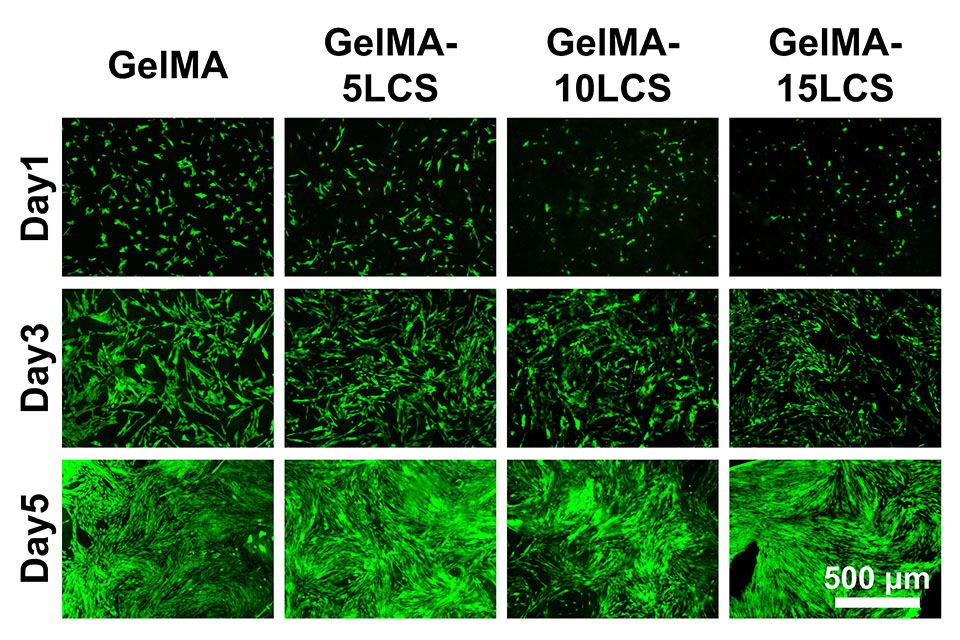


**Fig. S13** Live/dead staining images of dental pulp stem cells (DPSCs) cultured with composite hydrogels for 1, 3 and 5 days (Green: live cells; Red: dead cells).


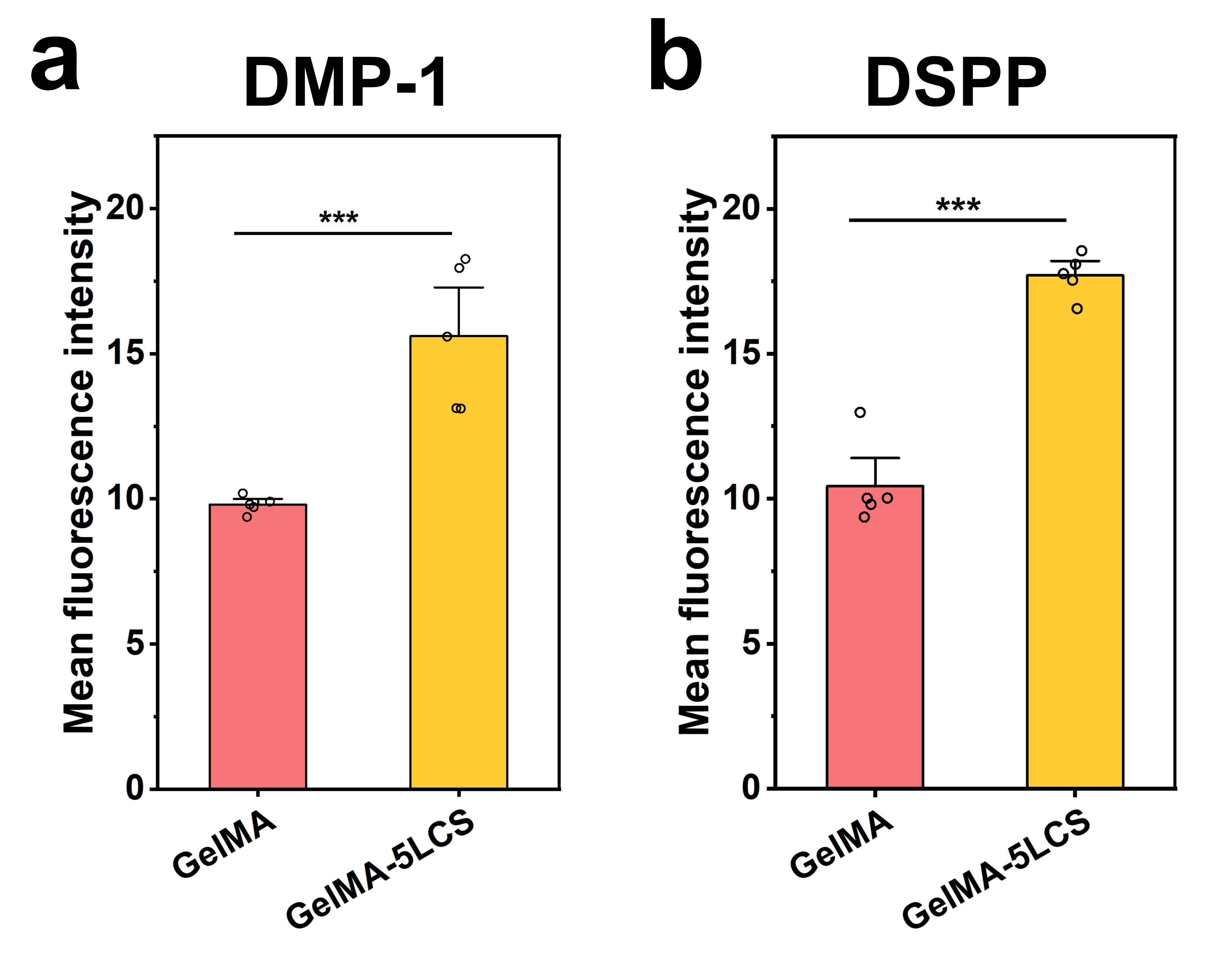


**Fig. S14** Semi-quantitative analysis of **a** DMP-1 and **b** DSPP proteins in DPSCs after cultured with GelMA and GelMA-5LCS hydrogel for 5 days (*n* = 5).


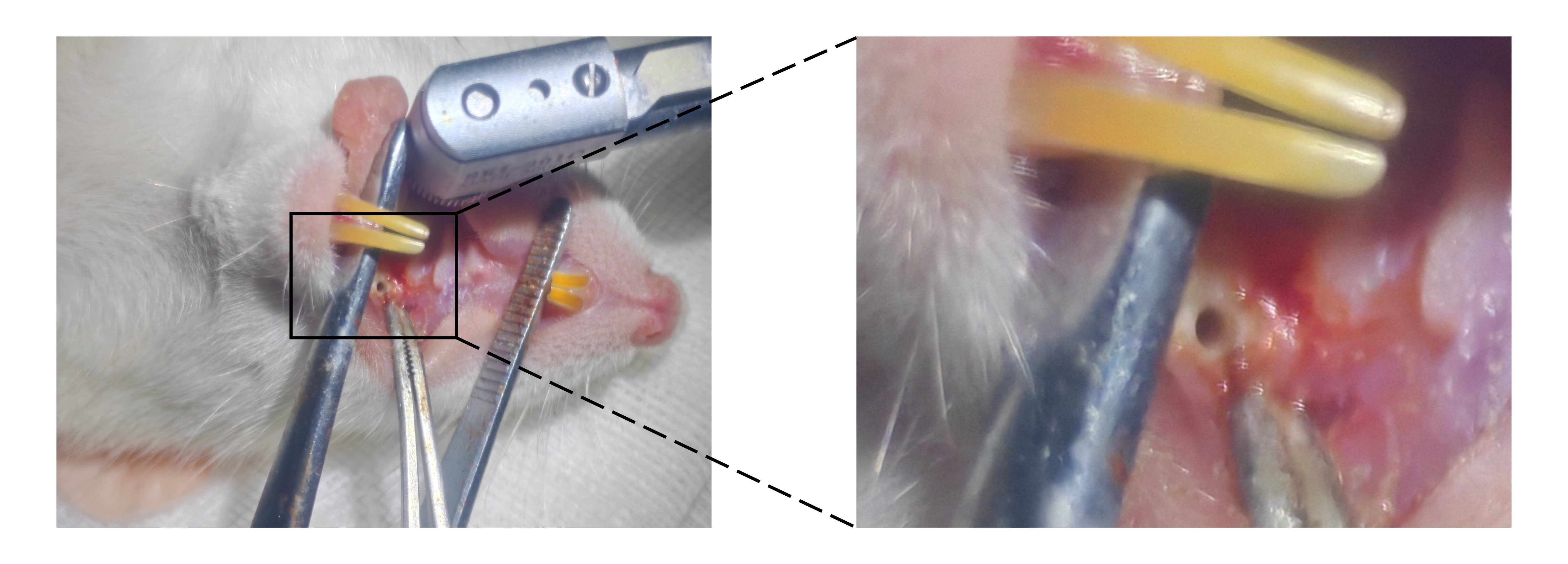


**Fig. S15** Images of the dentin-pulp defect established in the first molar teeth of rats.

**Table S1** The calcium, lithium and silicon ions release of composite hydrogels during the culture time. The ionic concentration of the culture medium at each time point (1, 3 and 5 days) (n = 3).

| **Ionic Conc**  **(mg L^-1^)** | **Time (d)** | **GelMA** | **GelMA-5LCS** | **GelMA-10LCS** | **GelMA-15LCS** |
| --- | --- | --- | --- | --- | --- |
| **Ca** | 1 | 55.09±0.57 | 59.42±0.19 | 57.76±1.21 | 62.23±0.83 |
|  | 3 | 57.67±1.41 | 62.09±1.29 | 62.83±0.68 | 59.18±1.00 |
|  | 5 | 60.51±0.38 | 60.03±0.56 | 60.22±1.58 | 62.07±7.62 |
| **Li** | 1 | 0.14±0.01 | 4.05±0.02 | 8.52±0.23 | 15.56±0.30 |
|  | 3 | 0.01±0.01 | 0.77±0.41 | 1.61±0.03 | 1.68±0.03 |
|  | 5 | 0.02±0.01 | 0.00±0.00 | 0.36±0.28 | 1.68±1.41 |
| **Si** | 1 | 0.89±0.07 | 13.59±0.21 | 22.32±0.32 | 37.48±0.48 |
|  | 3 | 0.90±0.05 | 4.06±0.98 | 5.65±0.16 | 15.96±0.14 |
|  | 5 | 0.14±0.14 | 0.83±0.03 | 4.75±1.33 | 11.95±2.92 |
| **Medium** | **Ca** | 65.54±0.20 | | | |
|  | **Li** | 0.01±0.00 | | | |
|  | **Si** | 0.05±0.05 | | | |

**Table S2** The primer sequences for RT-qPCR assays.

**Neurogenic-related genes**

| **gene** | **Primer sequences** |
| --- | --- |
| GAPDH | 5’-AGGTCGGTGTGAACGGATTTG-3’ (forward) |
| GAPDH | 3’-TGTAGACCATGTAGTTGAGGTCA-5’ (reverse) |
| GDNF | 5’-CTACGAAACCAAGGAGGAACTGA-3’ (forward) |
| GDNF | 5’-GGTAAACCAGGCTGTCGTCTAAA-3’ (forward) |
| PMP22 | 5’-GTCTGGTCTGCTGTGAGCAT-3’ (forward) |
| PMP22 | 3’-GCCATTGGCTGACGATGGTG-5’ (reverse) |
| NGF | 5’-TGATCGGCGTACAGGCAGA-3’ (forward) |
| NGF | 3’-GAGGGCTGTGTCAAGGGAAT-5’ (reverse) |
| BDNF | 5’-GTCAAGTGCCTTTGGAGCCT-3’ (forward) |
| BDNF | 3’-CTTATGAACCTTTGGAGCCT-5’ (reverse) |

**Odontogenesis-related genes**

| **gene** | **Primer sequences** |
| --- | --- |
| GAPDH | 5’-GGAGTCCACTGGCGTCTTCA-3’ (forward) |
| GAPDH | 5’-GTCATGAGTCCTTCCACGATACC-3’ (forward) |
| DMP-1 | 5’-GAGATAACCCCGACCCCACA-3’ (forward) |
| DMP-1 | 5’-GAGAGTGTGTGCGAGCTGTC-3’ (forward) |
| DSPP | 5’-GCTGGCCTGGATAATTCCGA-3’ (forward) |
| DSPP | 5’-CTCCTGGCCCTTGCTGTTAT-3’ (forward) |
| OPN | 5’-CAGTTGTCCCCACAGTAGACAC-3’ (forward) |
| OPN | 5’-GTGATGTCCTCGTCTGTAGCATC-3’ (forward) |
| ALP | 5’-ACATTCCCACGTCTTCACATTT-3’ (forward) |
| ALP | 5’-AGACATTCTCTCGTTCACCGCC-3’ (forward) |
